# Investigating the conservatism-disgust paradox in reactions to the COVID-19 pandemic: A reexamination of the interrelations among political ideology, disgust sensitivity, and pandemic response

**DOI:** 10.1371/journal.pone.0275440

**Published:** 2022-11-04

**Authors:** Benjamin C. Ruisch, Shelby T. Boggs, Courtney A. Moore, Javier A. Granados Samayoa, Jesse T. Ladanyi, Steffen Steinert, Russell H. Fazio

**Affiliations:** 1 University of Kent, Canterbury, United Kingdom; 2 The Ohio State University, Columbus, Ohio, United States of America; 3 Delft University of Technology, Delft, Netherlands; St John’s University, UNITED STATES

## Abstract

Research has documented robust associations between greater disgust sensitivity and (1) concerns about disease, and (2) political conservatism. However, the COVID-19 disease pandemic raised challenging questions about these associations. In particular, why have conservatives—despite their greater disgust sensitivity—exhibited *less* concern about the pandemic? Here, we investigate this “conservatism-disgust paradox” and address several outstanding theoretical questions regarding the interrelations among disgust sensitivity, ideology, and pandemic response. In four studies (*N* = 1,764), we identify several methodological and conceptual factors—in particular, an overreliance on self-report measures—that may have inflated the apparent associations among these constructs. Using non-self-report measures, we find evidence that disgust sensitivity may be a less potent predictor of disease avoidance than is typically assumed, and that ideological differences in disgust sensitivity may be amplified by self-report measures. These findings suggest that the true pattern of interrelations among these factors may be less “paradoxical” than is typically believed.

## Introduction

The emotion of disgust is believed to have evolved to facilitate disease prevention, motivating behavioral avoidance of situations and stimuli that hold the potential for pathogen transmission [1, 2]. It is theorized that early in human evolutionary history, disgust initially served specifically to prevent the ingestion of potentially toxic substances like rotten foods. However, this disgust response was subsequently generalized to be elicited by a myriad of potentially pathogenic stimuli. Consistent with this view, disgust in modern humans is triggered by many cues of potential contagion, such as bodily fluids (e.g., blood, vomit), certain insects and animals (e.g., cockroaches, rats), and humans exhibiting signs of illness (e.g., coughing, spasms, skin lesions [1–3]).

Disgust constitutes an integral part of the “behavioral immune system” (BIS), a suite of affective (e.g., the emotion of disgust), cognitive (e.g., disease-relevant thoughts), and behavioral (e.g., avoidance of disgusting stimuli) responses that collectively operate to limit the transmission of disease. The BIS operates in complement to the biological immune system. While the biological immune system eliminates pathogens after they have entered the body, the purpose of the BIS is to prevent these pathogens from entering the body in the first place. Disgust, and BIS reactivity more generally, is thus theorized to play an integral role in disease prevention by identifying and facilitating avoidance of stimuli that may pose a disease threat.

Despite the universality of the BIS and the emotion of disgust, however, people nonetheless exhibit substantial variability in their propensity to experience disgust, with some individuals generally showing greater sensitivity than others to disgusting situations and stimuli [1–3]. Those high in disgust sensitivity are more prone to perceiving even relatively benign stimuli as disgusting, they tend to experience the emotion of disgust more intensely, and they remain disgusted for a longer duration compared with those lower in sensitivity to disgust. Consistent with the theorized evolutionary function of disgust, individuals who are higher in disgust sensitivity also tend to exhibit greater concern about potentially contracting illnesses, greater sensitivity to cues or signals of disease, and greater behavioral avoidance of situations with the potential for pathogen transmission (e.g., contact with sick people [3–5]).

### Disgust sensitivity and political ideology

A large body of research has demonstrated that individuals who are dispositionally more disgust-sensitive tend to be more politically conservative, both in their self-identifications (i.e., symbolic ideology) and issue-positions (i.e., operational ideology) [e.g., 6, 7], as well as in their voting habits [8, 9]. This relationship between disgust sensitivity and conservatism tends to be strongest with respect to potential interpersonal contamination. That is, those who are more disgusted by interpersonal infection are most prone to holding conservative attitudes [8].

Providing tentative evidence for a causal relation between disgust and conservatism, research has also shown that situational manipulations of disgust (e.g., bad smells, disgusting images) can lead people to adopt more politically conservative positions, at least on certain issues (e.g., same-sex marriage [10]). Moreover, other research has documented ideological differences in physiological and anatomical traits that underpin sensitivity to disgust, such as taste sensitivity [11], suggesting that these ideological differences in disgust sensitivity may be rooted in relatively immutable elements of an individual’s biology.

Further supporting a possible causal connection between disgust sensitivity and ideology, other research has suggested that large-scale disease threats—which have been shown to increase disgust sensitivity [12]—may also lead to greater political conservatism. For example, disease salience during the 2014 Ebola outbreak was associated with greater intentions to vote for Republican candidates in the 2014 U.S. Federal elections [13]. More generally, past work has demonstrated that nations with higher levels of pathogen transmission tend to be more authoritarian and hold more traditional value systems, both of which are characteristic features of political conservatism [14–16].

The association between disgust sensitivity and ideology has been explained via several psychological mechanisms. In particular, leading theoretical perspectives contend that this relation is explained by the associations between dispositional disgust sensitivity and greater traditionalism and sexual restrictiveness [16, 17] and greater intergroup bias (i.e., favoritism toward social ingroups and derogation of social outgroups [7, 18]). According to these theoretical perspectives, individuals who are higher in disgust sensitivity tend to prefer groups with strong ingroup cohesion and rigid social norms, and they also tend to hold more prejudiced attitudes towards outgroup members. These preferences subsequently lead more disgust sensitive individuals to tend to adopt more politically conservative ideologies because conservatism better aligns with these positions and principles (e.g., by promoting traditional sexuality, limiting immigration, and providing greater ingroup protection).

Despite the large body of research on the association between disgust and conservatism, however, recent events have raised intriguing questions regarding the nature, extent, and importance of ideological differences in sensitivity to disgust. In particular, the COVID-19 disease pandemic—and ideological differences in responses to it—has revealed a pattern of effects that seems to challenge the idea that conservatives broadly exhibit greater sensitivity to disgusting situations and stimuli.

### The COVID-19 pandemic

The COVID-19 disease pandemic that began in late 2019 claimed hundreds of thousands of lives and exacted extensive economic and societal repercussions [19, 20]. Despite the profound and wide-ranging consequences of the pandemic, however, people vary greatly in their concern about the virus and their behavioral reactions to the pandemic. While some individuals express substantial worry about the virus and exhibit strict behavioral compliance with efforts to combat it (i.e. increased mask-wearing, adherence to social distancing guidelines, and increased hygiene behavior), other individuals have downplayed the importance of the pandemic and have fought against social distancing guidelines and other anti-virus measures [21–26].

Consistent with the theoretical framework outlined above, one predictor of responses to the pandemic are individual differences in disgust sensitivity. Several lines of research suggest that disgust sensitivity and broader BIS activation predict responses to the pandemic [e.g., 21, 27–30]. More disgust-sensitive individuals express greater attitudinal concern about the virus, more anxiety about contracting the virus, and self-report engaging in greater preventative health measures (e.g., social distancing, handwashing, mask-wearing) [21, 27–30].

Given conservatives’ well-established greater sensitivity to disgust, the existence of ideological differences in responses to the COVID-19 pandemic should come as no surprise. After all, the research and theory outlined above clearly suggest that conservatives should exhibit greater concern about the pandemic—and, indeed, about any large-scale disease threat. However, the direction of these ideological differences is actually in direct opposition to these predictions: Political conservatives—despite their greater sensitivity to disgust—do not exhibit greater concern about the virus, and, in fact, actually exhibit *less* concern than do liberals [23–26, 31, 32]. These ideological differences have been documented in polling with nationally representative samples [24–26], as well as social psychological research [e.g., 22, 23, 32]. Specifically, individuals who are more politically conservative report less frequent mask wearing, less compliance with CDC recommendations (e.g., stay-at-home orders & social distancing mandates), and reduced concerns about the severity of the pandemic [23–26, 31, 32].

Critically, these associations do not appear to be small or tenuous. Indeed, disgust sensitivity/BIS activation is argued to be among the most powerful predictors of responses to the virus [29]. The ideological gap in pandemic response is also quite substantial—for example, with more than double the number of right-leaning (68%) versus left-leaning (30%) individuals expressing the belief that the danger of the pandemic has generally been exaggerated [24]. The large size of these effects makes this conflicting pattern of results all the more difficult to explain.

### Investigating the conservatism-disgust paradox in pandemic response

In sum, then, disgust sensitivity, ideology, and pandemic response exhibit an intriguing—and seemingly somewhat paradoxical—pattern of interrelations: disgust sensitivity is reliably associated with political conservatism, and disgust sensitivity appears to be one of the most powerful predictors of concern about the virus. And yet, rather than being more concerned about the virus, conservatives are actually substantially less so.

How can we reconcile this pattern of effects? Why is it that disgust sensitivity is such a powerful predictor of responses to COVID-19, while the well-established ideological differences in disgust sensitivity appeared to have little impact in shaping conservatives’ and liberals’ responses to the pandemic? To be sure, there are many factors that shaped liberals’ and conservatives’ responses to COVID-19 beyond sensitivity to disgust, such as the “elite cues” emanating from Republican politicians that consistently downplayed the importance of the pandemic [33–35]. Nonetheless, the paradoxical pattern of relations among political ideology, disgust sensitivity, and responses to the pandemic is striking, and, we believed, deserving of closer examination. In this work, we therefore provide an in-depth re-examination of the interrelations among disgust sensitivity, ideology, and pandemic response, in the hopes of shedding further light on this seeming paradox.

In reviewing past research, we identified several factors that—independently or collectively—may help to account for the conservatism-disgust paradox in pandemic response. In particular, we identified three core conceptual and methodological issues that may have artificially amplified the apparent relations among these three factors (i.e., between disgust sensitivity and pandemic response; between disgust sensitivity and ideology; between ideology and pandemic response). If so, this may have contributed to this apparently paradoxical pattern of results. For example, if disgust sensitivity is in fact only a relatively weak predictor of pandemic response—or if ideological differences in disgust sensitivity are smaller than is typically believed—then it is less surprising that conservatives have exhibited so little concern about the virus, as these factors would have been more easily subsumed by other concerns and motivations, such as elite cues. Put another way, the degree of this apparent “paradox” may simply have been overstated.

**Fig 1:**
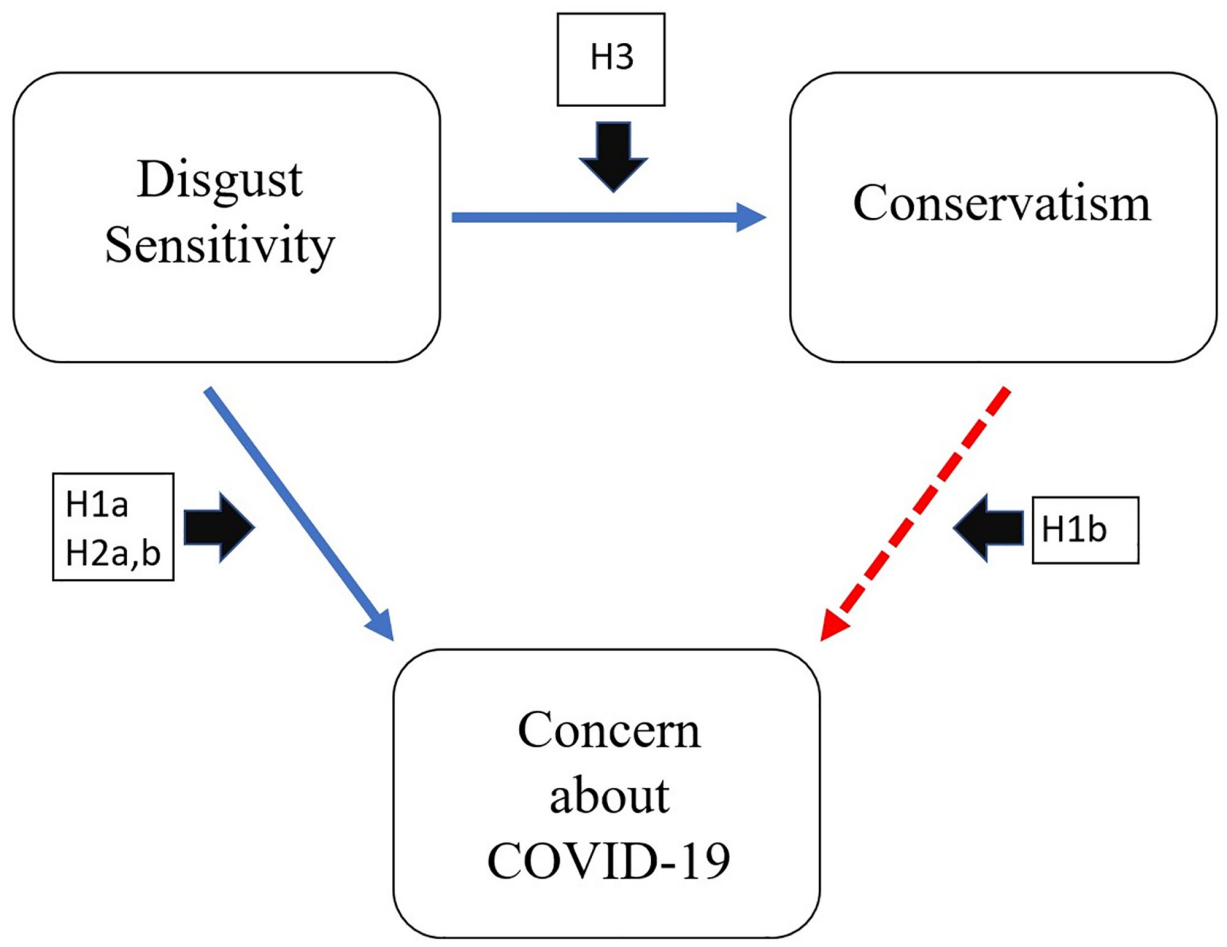
Model of the theorized interrelations among our three target variables, with hypotheses positioned according to the specific associations that they examine. Solid blue arrows indicate positive associations and dashed red arrows indicate negative associations.

Below, we briefly discuss each of these three possible explanations–(1) the use of self-report measures of pandemic response that are particularly susceptible to social desirability effects and other reporting biases, (2) content overlap between disgust sensitivity and pandemic response measures that artificially inflate the apparent strength of their association, and (3) overestimation of ideological differences in sensitivity to disgust through the use of self-report measures–and we present the specific hypotheses that we test to assess each explanation. Fig 1 outlines the nature of the hypothesized interrelations among each of our three factors of interest and visually represents which specific association is reexamined by each of our five hypotheses. These hypotheses, collectively, constitute a critical reexamination of the interrelations among disgust sensitivity, ideology, and pandemic response, providing novel insights into the nature of each “branch” of this triangular pattern of associations (each of which has independently been the subject of much research) as well as providing insight into the overarching question of what may explain the conservatism-disgust paradox in pandemic response.

#### Use of self-report measures of pandemic response

One possible contributing factor is that past research has predominately used relatively subjective self-report measures of pandemic response, such as attitude measures and self-reported behavior (e.g., social distancing, mask-wearing [27–31]). Such self-report measures are more susceptible to social-desirability motivations, self-presentational concerns, and other reporting biases [36–38], especially given the strong normative—and in some cases legal—pressure to engage in social distancing and other anti-virus behaviors [39, 40]. This may have amplified the apparent strength of the relations between pandemic response and disgust sensitivity. If true, then:

**H1a.** The relation between disgust sensitivity and pandemic response should be attenuated for behavioral (versus self-report) measures of pandemic response.

By the same token, the use of these subjective self-report measures of pandemic response may have artificially amplified the apparent strength of the association between ideology and concern about COVID-19. As noted above, responses to the pandemic were starkly polarized in the U.S. [23–26, 31, 32] with conflicting elite cues emanating from politicians on opposite sides of the political divide [33–35]. In particular, President Trump consistently and vocally downplayed the dangers of the pandemic [33, 34]. Past work has shown Trump’s rhetoric to have a particularly strong normative effect on self-reported attitudes, especially among conservatives [41], and there is evidence that Trump’s rhetoric about the pandemic strongly shaped both knowledge about [42] and responses to [22] the pandemic. Taken together, these factors may have led members of both ideological groups to exaggerate the degree to which their own attitudes and behaviors conformed to the signals sent by their respective elites. If true, then:

**H1b.** The relation between ideology and pandemic response should be attenuated for behavioral (versus self-report) measures of pandemic response.

#### Content overlap between disgust sensitivity and pandemic response measures

The self-report measures of disgust sensitivity/BIS activation that have been used in past research also suffer from a critical weakness in the current context: possible conceptual/content overlap with measures of pandemic response. For example, most past research on responses to COVID-19 [e.g., 27–30] has used the Perceived Vulnerability to Disease scale [43] as an index of BIS activation. This measure focuses largely on situations with clear and immediate relevance for disease transmission (e.g., being in close contact or proximity to sick individuals), and thus seems likely to capture behaviors that are intended to prevent the spread of COVID-19 specifically. For example, the items “I prefer to wash my hands pretty soon after shaking someone’s hand” and “It really bothers me when people sneeze without covering their mouths” could themselves be considered indicators of concern about COVID-19.

The Disgust Scale-Revised (DS-R [3, 44]), which has been most widely used in past research on ideological differences [7], may suffer a similar weakness. This measure assesses self-reported disgust and participants’ self-beliefs regarding how they would behave in a variety of hypothetical disgusting situations—at least some of which appear to directly relate to disease prevention/avoidance (e.g., “I probably would not go to my favorite restaurant if I found out that the cook had a cold”; “You take a sip of soda, and then realize that you drank from the glass that an acquaintance of yours had been drinking from”).

This conceptual overlap between IV and DV may have further inflated the apparent predictive power of disgust sensitivity for pandemic response. If this is true, then we should see asymmetries in the predictive power of disgust sensitivity measures that rely on self-reports/self-beliefs versus less reflective measures of disgust sensitivity—such as measures that rely on more immediate affective or “experiential” responses to disgusting stimuli (e.g., affective reactions to disgusting images). Specifically:

**H2a**. Self-report measures of pandemic response should be more strongly associated with self-report (versus experiential) measures of disgust sensitivity.

Accordingly, however, if this conceptual overlap is eliminated or reduced—such as via the use of experiential measures of disgust sensitivity and behavioral measures of pandemic response—then we may see that:

**H2b.** Experiential (versus self-report) measures of disgust sensitivity may relate more strongly to behavioral (versus self-report) measures of pandemic response.

#### Overestimation of ideological differences in sensitivity to disgust

Finally, another, perhaps more controversial, possibility is that the extent of ideological differences in disgust sensitivity may be overestimated. That is, although the disgust-conservatism association is supported by a large body of research, in recent years some commentators have argued against the idea that liberals and conservatives differ widely in their psychological traits and motivations [45–49, though see also 50, 51]. Such perspectives contend that methodological limitations of past research—especially the near-exclusive reliance on self-report measures of psychological motivations—has amplified the appearance of ideological differences [52, 53]. Such self-report measures of psychological motivations are argued to be more susceptible to factors such as self-presentational differences between liberals and conservatives, and conceptual overlap between measures of ideology and measures of psychological traits. Collectively, these confounding factors may have led past research to overestimate the degree of ideological differences in traits such as sensitivity to disgust.

These concerns are further underscored by recent failures to replicate some seemingly well-established ideological differences, including liberal-conservative differences in sensitivity to disgust. For example, in multiple well-powered studies, Bakker and colleagues [45] recently failed to find any evidence of ideological differences on non-self-report, experiential (in this case, physiological) measures of sensitivity to disgusting stimuli. Similarly, Osmundsen and colleagues [54], conducted two further replications of this effect, as well as reanalyzed all published findings on ideology and physiological reactions to threatening and disgusting images, and concluded that there was little evidence for ideological differences. (Importantly, though, these replication attempts did not include measures of attention, and there is some evidence that liberals and conservatives may nonetheless differ in their propensity to attend to threatening and disgusting stimuli [55]). In another line of work, Elad-Strenger, Proch, and Kessler [56] found that ideological differences in disgust sensitivity emerged on some self-report measures (e.g., the DS-R) but not others (e.g., a measure assessing self-beliefs about one’s frequency of experiencing disgust in everyday life, rather than reactions to specific disgusting stimuli as in the DS-R).

The above results also dovetail with other recent findings suggesting that people may have limited introspective ability into their own degree of sensitivity to disgust. Indeed, some research has found that self-reports of disgust sensitivity correlate only moderately—and sometimes not at all—with other, more indirect measures of responses to disgusting stimuli (e.g., more experiential measures such as image ratings, behavioral tasks [e.g., 18, 57, 58].

For these reasons, we also wished to examine whether ideological differences in disgust sensitivity might be overestimated by the self-report measures that are typically used in research. To test this question, we compared the relation of ideology with both self-reported disgust sensitivity, as well as to the more experiential measure of disgust sensitivity discussed above. If ideological differences in disgust sensitivity are indeed artificially amplified by self-report measures, then:

**H3.** Measures of disgust sensitivity that rely on more immediate experiential responses (versus self-reports of one’s sensitivity to disgust such as the DS-R) will show attenuated relations with ideology.

If this final hypothesis is correct, then the seemingly paradoxical pattern of results discussed above may stem in part from the simple fact that liberals and conservatives do not differ in their sensitivity to disgust to the degree suggested by past research. That is, if ideological differences in disgust sensitivity are in fact relatively small, then it is less surprising that they can be overridden by competing motivations (e.g., identification with one’s political ingroup and elite cues).

In sum, then, our core argument is that the true pattern of interrelations among these three factors is not, in fact, as paradoxical as is typically believed. Rather, we propose that methodological decisions of past research—particularly the tendency to rely on self-report measures—has artificially amplified the apparent strength of the (seemingly contradictory) relations among these constructs. If our predictions are correct, then these findings—though not quite “solving” the paradox *per se*—at least help to explain why ideological differences in sensitivity to disgust appear to have been so easily overridden by other competing motivations such as elite cues and identification with one’s ideological ingroup [cf. 59, 60].

## The present research

In this research, we provide an examination of the interrelations among disgust sensitivity, ideology, and pandemic response, testing these five hypotheses and answering the questions outlined above. To do so, we conducted a series of four studies (Total *N* = 1,764), using a range of measures and approaches, as well as reexamining data from a large-scale survey on the predictors of pandemic response. Key study design variables are shown in Table 1.

**Table 1:** Design for all studies.

Design Table				
Measures by Study and Sample*				
	Study 1	Study 2	Study 3	Study 4
Sample Size	299	901	300	152
Disgust				
DS-R Full scale	X			X
DS-R Contamination subscale		X		
Experiential disgusting Images			X	X
COVID-19				
Worry about contracting COVID-19	X	X	X	X
Likelihood of contracting COVID-19		X		X
Self-Reported social distancing	X	X		X
Concern about COVID-19		X		
Support of social distancing guidelines		X		
Economy Vs. COVID-19		X		X
Face Mask Use		X		
Self-reported quarantine		X		X
COVID-19 Exaggerated		X		
Close Contact				X
Behavioral Social Distancing		X		X
Political Ideology	X	X		X
Demographics				
Age	X	X		
Gender	X	X		
Race		X		
Income		X		
Education		X		

*All studies: Americans recruited via Mechanical Turk

In Study 1, we provided an initial examination of the interrelations among ideology, disgust sensitivity, and pandemic response. In this study, we used the DS-R as our measure of disgust sensitivity, allowing us to test the nature and strength of these relations using the measure of disgust sensitivity that has been most widely used in research on ideological differences.

In Study 2, we provided a more in-depth and nuanced examination of these relations. To do so, we re-examined data from a large-scale, multi-wave correlational study that we conducted to understand the predictors of responses to the pandemic [22]. Using these data, we examined whether and how this pattern of associations differs when using self-report (e.g., attitudes) measures of pandemic response, compared to more behavioral measures (interactive virtual social-distancing scenarios). In doing so, we test H1a and H1b—that the relations between pandemic response and both ideology and disgust sensitivity will be attenuated when behavioral measures are used.

In Study 3, we used a more experiential, non-self-belief-based measure of disgust sensitivity to determine whether this measure would also predict responses to the pandemic. In doing so, we provide a preliminary investigation of whether methodological issues such as conceptual overlap between self-report measures of disgust sensitivity and pandemic response may have inflated the apparent relations between these factors.

In Study 4, we assessed both self-reported disgust sensitivity (the DS-R) and the experiential disgust sensitivity measure from Study 3, allowing us to compare the predictive power of each measure for different dimensions of pandemic response (i.e., self-reports vs. behavior; H2a and H2b). We also tested the relation of each disgust sensitivity measure with ideology to gain insight into whether and to what degree ideological differences in disgust sensitivity may have been overestimated in past research (H3).

## Analytic approach and open science practices

We test our primary hypotheses using linear regression. For regression analyses, we report standardized beta coefficients. We list all predictors and control variables included in the models (if no covariates are stated, none were included). All participants who provided complete, analyzable data and passed our included attention checks are included in analyses. All materials, data, syntax, and preregistration documentation are available on the Open Science Framework at https://osf.io/3ypx2.

## Studies 1 and 2

In our first two studies, we provided an examination of the interrelations among ideology, disgust sensitivity, and pandemic response, using the measure of disgust sensitivity that has been most widely used in past research on ideological differences, the DS-R. In Study 1, we conducted a (preregistered) initial test of these associations. In Study 2, we provided a more in-depth examination of different dimensions of pandemic response, using a multi-faceted measure that included items assessing both attitudes and self-reported behavior. Critically, to overcome the limitations of these self-report measures, this study also employed an innovative behaviorally oriented measure of social distancing: virtual graphical scenarios in which participants interactively “distanced” themselves from others. These measures presented participants with several real-world scenarios and allowed them to position themselves relative to other people in the scene. This included situations such as distancing oneself from an oncoming walker, separating individuals waiting in line for the bus, and choosing a spot on a crowded beach. (Demo measures can be viewed at http://psychvault.org/social-distancing-measures/.) Critically, these behavioral measures have been shown to prospectively predict whether or not an individual contracts COVID-19—and even to be a more powerful predictor of subsequently contracting the virus than are self-report measures of social distancing [21]—thus providing an alternative, non-self-report-based measure of social distancing with greater predictive validity for contracting the virus.

### Method

This research received ethical approval from the Ohio State University Institutional Review Board under protocol #2020B0129. Informed consent was obtained from all participants.

#### Participants

We recruited participants from Amazon’s Mechanical Turk (MTurk) [61]. Although not a nationally representative sample, participants from MTurk are substantially more demographically, geographically, and—most importantly for the present purposes—ideologically diverse than the college student samples typically used in psychological research [62, 63]. They also perform similarly to non-MTurk samples across many tasks and measures, including measures of politically relevant beliefs and attitudes [62, 64]. Recruitment was limited to participants currently residing in the United States with an approval rate of at least 95%.

In Study 1, we recruited 300 participants. Consistent with our preregistered analysis plan (see OSF page), one participant was excluded from analyses for failing the included attention check, leaving a sample of 299 participants (134 female, 164 male, 1 nonbinary; *M*_age_ = 37.66; *SD*_age_ = 11.44).

In Study 2, we recruited a large sample to ensure we had sufficient power to probe higher-order interactions and provide more stable and accurate effect size estimates [65]. This study employed a planned-missing design [66] in which different subsets of participants were randomly assigned to complete different sets of measures in order to reduce participant fatigue. All participants completed our core survey which included six items assessing attitudes towards the pandemic (e.g., worry about COVID-19, perceptions that the threat is exaggerated, support for social distancing), five items assessing self-reported behavior (e.g., social distancing, mask wearing, handwashing), and our 10 virtual social-distancing measures. The core survey also included demographic questions and our measure of political orientation. Other measures, including disgust sensitivity, were administered to only subsets of the full sample. Two thousand and one people participated in the first wave of the study (May 7, 2020) and 1,508 participated in the second wave (June 9, 2020). (Study wave did not moderate our effects, so we simply combined both waves into a single dataset). Of these 3,509 participants, 1,010 were assigned to complete our target measures. Of these, 109 participants (10.8%) failed the included attention check, leaving an analyzable sample of 901 participants (404 female, 492 male, nonbinary; *M*_age_ = 38.77; *SD*_age_ = 12.41).

#### Procedure

The basic procedure of these two studies was similar. In Study 1, participants completed the 25-item DS-R (α = .88) and completed a 4-item measure of self-reported pandemic response (α = .80) that included two questions assessing attitudes towards the pandemic (e.g., “Generally speaking, how worried are you that you personally will contract COVID-19 / the coronavirus?”) and two questions assessing self-reported behavior (e.g., “Generally speaking, how strictly have you personally been following the ‘social distancing’ recommendations of the government and CDC to maintain a distance of six feet or more from others?”). Participants then indicated their political ideology (*M* = 3.31, *SD* = 1.77) using a 7-point scale ranging from “Extremely Liberal” to “Extremely Conservative”, with the midpoint labeled “Neither/Moderate” (e.g., [67]) and then reported their age and gender.

In Study 2, participants first completed our multi-faceted measure of pandemic response, which included six items assessing attitudes towards the virus (e.g., belief that the threat is exaggerated; α = .72; See Supplemental Materials (SM) for all measures), four items assessing self-reported behavior (e.g., mask-wearing, handwashing; α = .70), and ten virtual behavioral distancing scenarios, as described above (α = .81). Participants then completed a shortened version of the DS-R (the 5-item contamination subscale; α = .70). They then responded to some other measures (see SM for all measures), provided demographic information, and indicated their ideology using the same scale from Study 1 (*M* = 3.60; *SD* = 1.81).

### Results

Study 1 confirmed the paradoxical pattern of results that we anticipated based on past research. We found that greater disgust sensitivity (as assessed by the DS-R) was associated with greater political conservatism (β = .17, *t*(297) = 2.99, *p* = .003, 95% CI[.06, .28]), and we also found that disgust sensitivity predicted greater concern about the pandemic (β = .20, *t*(297) = 3.59, *p* < .001, 95% CI[.09, .32]). Consistent with past research concerning the pandemic, we also found that more conservative (versus liberal) participants exhibited substantially less concern about the virus (β = -.32, *t*(297) = 5.81, *p* < .001, 95% CI[-.43,-.21]).

Study 2 replicated this general pattern of results, while extending these findings in several important ways. To assess the overall pattern of associations among these variables, we first created a composite measure of overall pandemic response by combining the attitudes, self-reported behavior, and behavioral distancing dimensions (α = .80). Using this measure, we replicated the results of Study 1: disgust sensitivity (as assessed by the DS-R) was positively associated with conservatism (β = .18, *t*(896) = 5.38, *p* < .001, 95% CI[.11, .24]) and with pandemic response (β = .24, *t*(898) = 7.44, *p* < .001, 95% CI[.18, .30]). As in Study 1, ideology was negatively associated with pandemic response (β = -.33, *t*(897) = 10.43, *p* < .001, 95% CI[-.39,-.27]), such that conservatives expressed considerably less concern about the virus. (Means, Standard Deviations and Correlations for all Study 2 variables are displayed in Table 2). Notably, the effect sizes for all of these relations were remarkably similar to those observed in Study 1, with βs/*r*s deviating only by .01, .04, and .01, respectively.

**Table 2:** Means, standard deviations, and correlations for all Study 2 variables.

	1	2	3	4	5
1. DS-R Contamination Subscale	1.00				
2. Political Ideology	0.18***	1.00			
3. Behavioral Distancing	0.13***	-0.22***	1.00		
4. Self-Reported Behavior	0.24***	-0.25***	0.54***	1.00	
5. Self-Reported Attitudes	0.23***	-0.36***	0.50***	.66***	1.00
Mean	3.14	3.60	0.03	0.04	-0.01
SD	0.89	1.81	0.57	0.75	0.66

*Note*. Self-reported attitudes is an average of six items assessing attitudes towards the pandemic (e.g., worry about COVID-19, perceptions that the threat is exaggerated, support for social distancing), Self-reported behavior is an average of five items assessing behaviors during the pandemic (e.g., social distancing, mask wearing, handwashing), and Behavioral distancing is an average of 10 virtual social-distancing measures.

** indicates *p* < .01,

*** indicates *p* < .001

We next tested our first set of hypotheses by comparing self-reported pandemic response (attitudes and self-reported behavior) to our behavioral measure (the virtual social-distancing scenarios). Supporting H1a, we found that the strength of the relation between disgust sensitivity and pandemic response differed as a function of measure type (*F*(1,898) = 23.96, *p* < .001), with disgust sensitivity showing a stronger relation to self-reported pandemic response (β = .26, *t*(898) = 7.99, *p* < .001, 95% CI[.19, .32]), but only a weak association with the behavioral measures of pandemic response (β = .13, *t*(898) = 3.91, *p* < .001, 95% CI[.06, .19]).

Similarly, supporting H1b, we also found that the strength of the association between ideology and pandemic response significantly differed as a function of measure type (*F*(1,897) = 22.59, *p* < .001), with political ideology showing a stronger association with self-reports of pandemic response (β = -.33, *t*(897) = 10.58, *p* < .001, 95% CI[-.40,-.27]) than with behavioral responses (β = -.22, *t*(897) = 6.69, *p* < .001, 95% CI[-.28,-.15]). In exploratory analyses, we further subdivided the self-report measures into separate indices for attitudes and self-reported behavior. We found that ideological differences were descriptively larger for attitudes (β = -.36, *t*(897) = -11.57, *p* < .001, 95% CI[-.42,-.30]) than for self-reported behavior (β = -.25, *t*(897) = -7.82, *p* < .001, 95% CI[-.32,-.19]), consistent with the possibility that identity-related concerns may amplify the appearance of ideological differences in pandemic response.

### Discussion

The results of Studies 1 and 2 provided clear support for our predictions. First, we replicated the seemingly paradoxical pattern of interrelations that we anticipated based on past work: Disgust sensitivity positively predicted both conservatism and concern about COVID-19, and yet, conservatives—despite their greater sensitivity to disgust—nonetheless exhibited substantially less concern about the virus.

Importantly, however, these results also provided novel insights into the nature and extent of these relations. First, we found that the effect sizes using the DS-R measure of disgust sensitivity—which has been favored in research on ideological differences, but which has been rarely (if at all) used in research on COVID-19—were relatively small. This was true both of the relation between disgust sensitivity and pandemic response, as well as the relation between disgust sensitivity and ideology, both of which exhibited associations of approximately *r*/β = .2.

Second, we also identified a factor that is likely to have inflated the strength of the relations among these variables in past research: the exclusive use of self-report measures of pandemic response. Using an alternative, virtual behavioral measure of social distancing, we observed attenuated relations between ideology and concern about the pandemic, such that conservatives and liberals differed substantially less in their behavior in pandemic-relevant situations than they did in their attitudes and self-reported behavior regarding the pandemic (H1b).

Similarly, these behavioral measures of pandemic response also exhibited an attenuated relation to disgust sensitivity, compared with self-report pandemic response measures (H1a). This pattern is consistent with the possibility of IV-DV content/conceptual overlap in past research, which may have inflated the apparent relation between disgust sensitivity/BIS activation and pandemic response.

In sum, then, the paradoxical pattern of relations among ideology, disgust sensitivity, and pandemic response emerged in these studies as well, although our findings suggest that the extent/strength of the interrelations among these variables is likely to have been amplified by methodological characteristics of past research. These findings therefore take us one step closer to understanding the conservatism-disgust paradox in pandemic response, by suggesting that the true pattern of results is not as paradoxical as previously believed. That is, given that these relations appear to be somewhat smaller than has been suggested by past research, it is less surprising that conservatives’ higher sensitivity to disgust may not have led them to exhibit greater concern about COVID-19.

Importantly, however, even using this modified set of measures (e.g., the DS-R to assess disgust sensitivity; self-report measures of pandemic response), this paradoxical pattern did nonetheless continue to emerge. This suggests that these factors alone are unlikely to wholly account for the conservatism-disgust paradox. In Studies 3 and 4, we therefore continued to examine other factors that may have contributed to this pattern of results.

## Study 3

In Study 3, we wished to examine whether a more experientially-based measure of sensitivity to disgust—ratings of disgusting images—would also be associated with pandemic response. To create this experiential measure of disgust sensitivity, we created a visual analogue to the DS-R scale–one that relies less on participants’ self-reports and self-beliefs about their own sensitivity to disgust, and more on their immediate experiential reactions to disgusting stimuli. For this measure, we chose images that visually represented four vignettes from the DS-R scale. Specifically, the item “You see maggots on a piece of meat in an outdoor garbage pail” was represented by an image of maggots on a piece of meat; “It would not upset me at all to watch a person with a glass eye take the eye out of the socket” (reverse-scored) was represented by an image of a person taking a glass eye out of the socket; “It would bother me to be in a science class, and to see a human hand preserved in a jar” was represented by an image of a human hand preserved in a jar; and “You see someone put ketchup on vanilla ice cream, and eat it” was represented by an image of a person putting ketchup on vanilla ice cream. Participants were asked to rate the degree of disgust that they experienced upon viewing these four images.

### Method

#### Participants

We recruited 300 participants from Mechanical Turk. (No demographic information was collected.)

#### Procedure

Participants first completed our experiential measure of disgust sensitivity, which consisted of the four images described above. They rated each image on an 11-point scale from 0 “Not disgusting at all” to 10 “Extremely disgusting” (mean ratings of the disgusting images = 7.90, *SD* = 1.85, α = .57). For exploratory purposes, they also rated one milder, more ambiguously disgusting image (a birthday cake in the shape of a cockroach), and as control variables/covariates, they rated one positive image (a puppy), and one neutral image (a stapler).

Participants then indicated their concern about the pandemic. For this measure, we chose the item that showed the highest item-total correlation (.58) in Study 2, which assesses general worry about the pandemic: “Generally speaking, how worried are you that you personally will contract COVID-19 / the coronavirus?” (Because our predictions for this study did not involve political ideology, we did not assess ideology in this study.) Additionally, we randomly assigned participants to read either that the survey was about (1) everyday life, or (2) the COVID-19 pandemic, in case this affected the nature of the relations between disgust sensitivity and pandemic response. However, this manipulation had no effect on participants’ responses (*p*s > .29), and it is therefore not discussed further.

### Results

As predicted, we found that this more experiential measure of disgust sensitivity was positively associated with the pandemic response measure, i.e., worry about contracting COVID-19 (β = .13, *t*(295) = 2.22, *p* = .03, 95% CI[.02, .24]). Ratings of the ambiguously disgusting image were also significantly related to pandemic response (β = .19, *t*(295) = 3.28, *p* = .001, 95% CI[.08, .30]). The relation between our experiential disgust measure and pandemic response also remained significant when adjusting for ratings of the control images (β = .11, *t*(294) = 2.05, *p* = .04, 95% CI[.004, .22]).

### Discussion

These results conceptually replicate those of past work and our previous studies, showing that sensitivity to disgust—in this case, assessed by a more experiential and less self-report-based measure—is significantly associated with responses to the pandemic. This finding provides important convergent support for the existence of a connection between disgust sensitivity and pandemic response by showing that this association extends beyond self-reported disgust. Notably, however, the strength of the disgust-pandemic response association in this study was relatively weak and, at least descriptively, was smaller in size than that observed using self-reported disgust sensitivity in our previous studies, consistent with the possibility that such self-report measures may artificially amplify the apparent strength of the relation between disgust sensitivity and pandemic response (e.g., because of IV-DV conceptual overlap). In Study 4, we test this question more directly by contrasting the predictive power of self-report and experiential disgust sensitivity measures.

## Study 4

In this study, we had two primary aims. First, we directly compared the pattern of relations using our experiential measure of disgust sensitivity to self-reported disgust sensitivity, the DS-R. Given possible conceptual overlap between self-report measures of disgust sensitivity and self-report measures of pandemic response, we predicted that the DS-R would relate more strongly to self-report pandemic response measures (H2a). Critically, however, we predicted that the more experiential measure of disgust sensitivity would relate more strongly to behavioral measures of pandemic response (H2b).

Second, we also wished to test whether the apparent extent of ideological differences in disgust sensitivity may be amplified by the use of self-report measures. If so, then our experiential measure of disgust sensitivity (compared with the DS-R) should show attenuated relations with conservatism (H3). If this is true, this may suggest an additional possible reason why conservatives’ previously documented higher sensitivity to disgust did not translate into greater concern about the COVID-19 pandemic: The degree of these ideological differences may have been overestimated.

### Method

#### Participants

One hundred and fifty-four participants completed this survey. Two participants did not complete our pandemic response measure or indicate their political ideology and could therefore not be included in analyses, leaving a sample of 152 (65 female, 86 male, 1 nonbinary; *M*_age_ = 37.78; *SD*_age_ = 11.18). (These participants were also surveyed previously, in November 2019, as part of a larger sample of 251 participants. Save for our experiential disgust and COVID-19-related measures, this previous survey was identical to the one described here.)

#### Procedure

Participants first completed some measures unrelated to the present hypotheses (see OSF page for all measures). They then completed the full DS-R and a shortened version of our multi-faceted pandemic response measure, which included three items assessing attitudes (worry about contracting the virus, perceived likelihood of contracting the virus, and belief that the government should prioritize controlling the virus), two assessing self-reported behavior (number of people with whom they had close contact, self-reported social distancing), and two virtual behavioral scenarios (grocery shopping and walking with a friend). They then completed the experiential measure of disgust sensitivity from Study 3, consisting of the same four images derived from the DS-R vignettes (α = .68). As in Study 3, for exploratory purposes participants also rated four milder disgusting images *not* taken from the DS-R (e.g., a birthday cake in the shape of a raw chicken). As control variables/covariates, they also rated four positive images. Finally, they reported their political ideology on a 9-point scale (*M* = 4.31, *SD* = 2.52), provided demographic information, and answered a few additional questions unrelated to the present hypotheses (see OSF page).

### Results

As in our previous studies, we first examined the pattern of relations using the composite measure of pandemic response. Replicating our previous results, we found a significant association between self-reported disgust sensitivity (i.e., DS-R scores) and political conservatism (β = .17, *t*(150) = 2.14, *p* = .03, 95% CI[.01, .33]), a marginally significant association between self-reported disgust sensitivity and pandemic response (β = .16, *t*(150) = 1.95, *p* = .053, 95% CI[-.002, .32]), and a negative association between conservatism and pandemic response (β = -.22, *t*(150) = -2.75, *p* = .007, 95% CI[-.38,-.06]).

We next turned to our experiential measure of disgust sensitivity. We first examined the strength of the association with self-reported disgust sensitivity/DS-R scores. Although the self-report and experiential measures were positively correlated, the association was nonetheless relatively modest in size (*r* = .36, *p* < .001). Even more strikingly, the same modest correlation was true even when comparing experiential disgust scores to ratings of the four specific DS-R vignettes on which the experiential disgust images were based (*r* = .28, *p* < .001). These results appear consistent with past work showing that self-reported disgust sensitivity often correlates only moderately with other, more indirect or behavioral measures of sensitivity to disgust [18, 57, 58].

We next compared the overall predictive power of these two disgust sensitivity measures for our composite measure of pandemic response. Interestingly, we found that the relation between our experiential disgust sensitivity measure and responses to the pandemic (β = .26, *t*(150) = 3.30, *p* = .001, 95% CI[.10, .42]) was significantly stronger than the association between self-reported disgust sensitivity and pandemic response (β = .16; difference: *F*(1,150) = 6.35, *p* = .01). Even more surprisingly, we found that when both measures were included in the model, *only experiential disgust significantly predicted responses to the pandemic* (β = .23, *t*(149) = 2.76, *p* = .007, 95% CI[.06, .40]); self-reported disgust sensitivity was not significant (β = .07, *t*(149) = 0.85, *p* = .40, 95% CI[-.10, .24]).

To provide an even more stringent test of the predictive power of experiential versus self-reported disgust sensitivity, we also conducted a separate set of analyses in which we compared our experiential disgust sensitivity measure to participants’ ratings of *the four specific DS-R vignettes on which the experiential disgust images were based* (rather than the full DS-R scale). In effect, this allowed us to test the relative predictive power of ratings of the same stimuli, varying only the way in which they were presented/rated: experiential responses to images versus self-reported responses to the vignettes. Notably, the pattern of results using these measures was nearly identical to that described above. With both measures in the model, only experiential disgust was a significant predictor of responses to the pandemic (β = .24, *t*(149) = 2.90, *p* = .004, 95% CI[.08, .40]), while self-reported disgust was not significant (β = .08, *t*(149) = 0.91, *p* = .37, 95% CI[-.09, .24]).

To better understand these associations, we next tested H2a and H2b by comparing the pattern of effects with our experiential and self-report disgust sensitivity measures with different dimensions of pandemic response—both self-report and behavioral measures. Here we found that experiential disgust sensitivity was a better predictor of both behavioral *and* self-reported pandemic response. With both disgust measures in the model, only *experiential* disgust sensitivity predicted *behavioral* pandemic response (β = .23, *t*(149) = 2.74, *p* = .007, 95% CI[.07, .40]), while self-reported disgust sensitivity was non-significant (β = .03, *t*(149) = 0.40, *p* = .69, 95% CI[-.13, .20]; supporting H2b). Similarly, with both disgust measures in the model, *self-reported* pandemic response exhibited a stronger relation to *experiential* disgust sensitivity (β = .16, *t*(149) = 1.81, *p* = .07, 95% CI[-.01, .33]) than it did to self-reported disgust sensitivity (β = .08, *t*(149) = 0.90, *p* = .37, 95% CI[-.09, .25]), opposing H2a.

**Fig 2:**
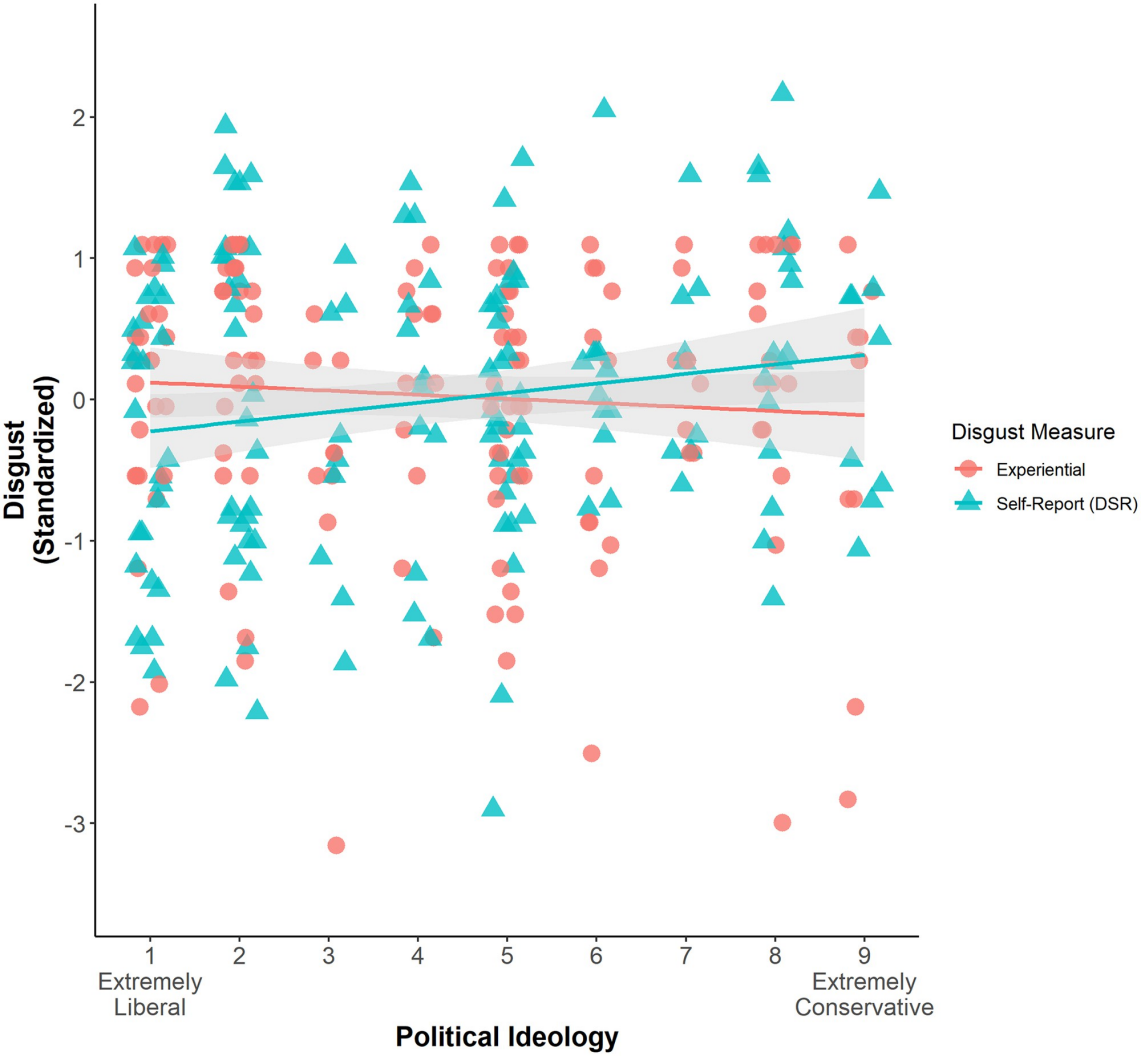
Scatterplot illustrating the relations between political ideology and the two disgust sensitivity measures from Study 4. Experiential disgust sensitivity scores are plotted as red circles, and self-reported disgust sensitivity scores are plotted as green triangles. Shaded areas represent the 95% confidence intervals of the regression lines.

Finally, we examined H3, that political ideology would show an attenuated relation to experiential (versus self-reported—i.e., the DS-R) disgust sensitivity. Supporting this hypothesis, we found a significant interaction between ideology and measure type (*F*(1,150) = 5.72, *p* = .02). As noted above, there was a significant association between DS-R scores and political conservatism (β/*r* = .17, *t*(150) = 2.14, *p* = .03, 95% CI[.01, .33]), consistent with much previous research [7]. However, with the experiential measure of disgust sensitivity, this relation was not only attenuated, but we actually found *no association between conservatism and experiential disgust whatsoever* (and the relation was in fact descriptively in the opposite direction, β/*r* = -.10, *t*(150) = -1.24, *p* = .22, 95% CI[-.26, .06]; Fig 2). Thus, on the experiential disgust sensitivity measure—which was also the better predictor of pandemic response—there were no meaningful ideological differences.

Importantly, the absence of a relation between ideology and experiential disgust sensitivity was not due to the specific vignettes that we selected to represent visually for our experiential measure: the same divergence was clear even when comparing participants’ ratings of the four specific target DSR vignettes to their ratings of the four images representing the same content (repeated measures ANOVA: *F*(1,150) = 4.17, *p* = .04).

To further assess the generalizability of this (lack of an) effect, we also examined the relation between ideology and ratings of the milder disgusting images. For these images, too, we found no relation whatsoever between ideology and experienced disgust (β = -.02, *t*(150) = -0.19, *p* = .85, 95% CI[-.18, .15]). These findings suggest that the weaker—or perhaps even nonexistent—ideological differences on more experiential measures of disgust sensitivity extend beyond the specific images that we selected from the DSR.

### Discussion

Our fourth and final study provided important new insights into the conservatism-disgust paradox, as well as into the interrelations among each of these factors. The pattern of results with our experiential and self-report disgust sensitivity measures were particularly intriguing. We found that experiential disgust sensitivity was a stronger predictor of pandemic response than was self-reported disgust sensitivity (H2a and H2b)—and that when both measures were in the model, only experiential disgust significantly predicted responses to the pandemic. Thus, even in spite of factors such as possible conceptual overlap between self-report disgust sensitivity and pandemic response measures, it was nonetheless experiential disgust sensitivity that proved to be the more powerful predictor of responses to the pandemic. These results provide some reason to be skeptical of self-report measures of disgust sensitivity in the context of the COVID-19 pandemic.

Further, we found support for the prediction (H3) that ideological differences in disgust sensitivity would be attenuated on an experiential measure of disgust sensitivity, compared with self-reports. These results appear consistent with recent critiques arguing that overreliance on self-report measures of psychological traits may have amplified the appearance of ideological differences in traits such as sensitivity to disgust. Indeed, using this more experiential measure we found no evidence whatsoever that conservatives are more sensitive to disgust.

Considered together, these results suggest an additional, and relatively straightforward reason why previously-observed ideological differences in disgust sensitivity may have failed to translate into commensurate responses to the COVID-19 pandemic: The extent of these differences may simply be overestimated. At the very least, these results seem to suggest that the form of disgust sensitivity in which liberals and conservatives most strongly differ may be less relevant for pandemic response. On a more experientially based assessment of sensitivity to disgust—the kind that mattered most for pandemic response—we observed no ideological differences.

## General discussion

This research provides important insight into the conservatism-disgust paradox in responses to the pandemic, as well as the relations among each of these target constructs—disgust sensitivity, political ideology, and pandemic response. These studies identified multiple factors that influence the (apparent) strength of the relations among these variables, thereby pinpointing several factors that are likely to have contributed to this seemingly contradictory pattern of results (Table 3).

**Table 3:** List of hypotheses, the study in which each hypothesis was tested, and whether or not each hypothesis was supported.

#	Hypothesis	Study	Supported
H1a	The disgust-pandemic response relation will be weaker for behavioral (vs. self-report) measures of pandemic response.	S2	Yes
H1b	The ideology-pandemic response relation will be weaker for behavioral (vs. self-report) measures of pandemic response.	S2	Yes
H2a	Self-report (vs. experiential) measures of disgust sensitivity will relate more strongly to self-report measures of pandemic response.	S4	Mixed
H2b	Experiential (vs. self-report) measures of disgust sensitivity will relate more strongly to behavioral measures of pandemic response.	S4	Yes
H3	Experiential (versus self-report) measures of disgust sensitivity will show an attenuated relation with ideology.	S4	Yes

One contributing factor appears to be the predominant use of self-report measures of pandemic response in past research. Indeed, using a behavioral measure of virtual social distancing, we found that the relations between pandemic response and both ideology and disgust sensitivity were significantly attenuated, compared with self-report pandemic response measures. These findings are consistent with the possibility that these self-report measures may suffer from IV-DV conceptual overlap, while also being more susceptible to social desirability and other reporting biases [36–38]. Particularly given that this same virtual behavioral measure has been shown to out-predict self-reports in predicting who contracts the COVID-19 virus [21], these results suggest that behavioral measures of pandemic response may provide a more accurate estimate of the extent of ideological differences in responses to the COVID-19 pandemic, as well as of the predictive power of disgust sensitivity for pandemic response. We found a similar divergence between self-report and non-self-report measures in the domain of disgust sensitivity. In this case, however, it was our experiential measure of disgust sensitivity that was the more powerful predictor of pandemic response. These findings identify important additional caveats and considerations for research examining the impact of disgust sensitivity on real-world outcomes, suggesting, in line with some past research, that self-reports of disgust sensitivity may correlate only modestly with other, more experiential or indirect indices of sensitivity to disgust—and that these measures/operationalizations may have different predictive power for different kinds of attitudes and behavior.

These findings also provide a means of beginning to reconcile some of the puzzling associations uncovered in other research on the COVID-19 pandemic. In particular, recent work suggests that—despite the putative disease-protective function of disgust—individuals who scored higher on self-reported disgust sensitivity may actually have been *more* likely to contract COVID-19 than those who self-reported less disgust sensitivity [23]. As documented here, however, self-reported disgust sensitivity appears to be only a relatively weak predictor of behavioral responses to the pandemic (indeed, adjusting for our experiential disgust measure rendered this association effectively nonexistent). Thus, although questions remain, these findings may bring us a step closer to understanding how self-reported disgust sensitivity could be a positive predictor of contracting the COVID-19 virus.

Perhaps the most intriguing findings, however, concern the relation of political ideology to self-report and experiential measures of disgust sensitivity. Using the DS-R, we replicated the well-documented ideological differences in self-reported disgust sensitivity. However, using our more experiential measure of disgust sensitivity—which presented participants with visual stimuli that closely corresponded to those described in the DS-R vignettes—we found no evidence of liberal-conservative differences in sensitivity to disgust.

Taken together, the findings discussed above suggest that methodological features of past research—particularly the heavy reliance on self-report measures of disgust sensitivity and pandemic response—may have inflated the relations among these three variables, and, thus, contributed to this seemingly contradictory pattern of results. In identifying the influence of these methodological factors, this research brings us a step closer to resolving the conservatism-disgust paradox, suggesting that the true pattern of interrelations among these variables is not as “paradoxical” as is typically assumed. That is, if, as these findings suggest, (1) the true relation between disgust sensitivity and pandemic response is smaller than previously suggested, and (2) ideological differences in disgust sensitivity are overestimated, then it is less surprising that conservatives exhibit less concern about the virus—particularly given that (3) ideological differences in responses to the pandemic may not be as dramatic as has been suggested by past research. The relatively small size of these effects makes it more likely that they would be subsumed by other concerns and motivations such as ideological identification and elite cues.

More generally, these findings also pose some challenges for past research and theory—particularly work suggesting a general relation between disgust sensitivity and political ideology. At the very least, these findings appear to suggest that liberals and conservatives do not differ in the form of disgust sensitivity that is most predictive of pandemic response. A more pessimistic interpretation, however, is that ideological differences in disgust sensitivity may generally be overestimated. That is, consistent with some recent critiques, it may be that self-report measures such as the DS-R amplify the true degree of ideological differences in disgust sensitivity, at least compared with measures that rely less on self-reports and self-beliefs about one’s own sensitivity to disgust.

Of course, our findings stand in contrast to a large body of research that suggests a connection between ideology and disgust, and, clearly, liberals and conservatives *do* reliably differ on many measures of disgust sensitivity (in particular, the DS-R and similar vignette-based measures). However, our findings also seem to align with other recent failures to replicate ideological differences in sensitivity to disgust using more indirect or experiential measures (e.g., [45]). Particularly in light of other research suggesting that people may have limited introspective ability into their own level of disgust sensitivity (e.g., work showing that self-reports sometimes do not significantly correlate with more indirect measures of disgust sensitivity; e.g., [18, 57, 58]) a closer examination of the nature and extent of ideological differences in disgust sensitivity may be warranted.

These findings therefore suggest that there may be a theoretical gap in our understanding of the relation between ideology and disgust sensitivity: Why is it that ideological differences reliably emerge on some measures of disgust sensitivity (e.g., the DS-R) but not others—even, as we found, measures that assess responses to closely related, or even identical, situations and stimuli? One possibility is that the ideological differences on the DS-R and similar vignette-based measures of sensitivity to disgust can in part be attributed to factors other than disgust sensitivity *per se*.

For example, forthcoming research suggests that conservatives tend to self-report greater interoceptive sensitivity—that is, to subjectively feel that they are more sensitive to the internal physiological states and signals of their own bodies—although by objective metrics they are actually *less* sensitive than are liberals [68]. Moreover, other research suggests that conservatives’ overconfidence may extend beyond interoception to experiences, judgments, and perceptions writ large [69]. Extending these past findings to the domain of disgust sensitivity would seem to suggest that conservatives may be likely to subjectively feel that they are more sensitive to disgust than they actually are, perhaps explaining why self-report measures of disgust sensitivity—which in part assess self-beliefs about one’s own degree of sensitivity to disgust—show more robust associations with conservatism than measures of disgust that are rooted in more immediate experience.

Less interestingly, another potential explanation for the weaker relation between ideology and our experiential disgust measure may be that previously documented ideological differences in personality traits such as conscientiousness [70] lead conservatives to complete survey measures more thoughtfully, perhaps reading more carefully or engaging more deeply with the material. This, too, could help explain why conservatives report experiencing greater disgust in response to these vignettes—which require a degree of cognitive effort to process and mentally represent—but do not appear to differ as greatly when these same stimuli are presented visually. Future research may wish to assess these possibilities to deepen our understanding of the nature of the relation between ideology and sensitivity to disgust.

More generally, these findings suggest that caution may be warranted in the development and use of measures to assess these constructs—disgust sensitivity, political ideology, and pandemic response—and, especially, their interrelations. Given the close connections among these factors, coupled with potential confounds such as self-presentational concerns that may be at play for such impactful and politicized issues as the COVID-19 pandemic, the use of self-report measures, in particular, should be subject to close scrutiny.

Finally, it is important to note that while our studies consistently show that using self-report scales may overestimate the strength of the interrelations among disgust sensitivity, pandemic response, and political ideology, some of these effects may be specific to the population that we sampled. Indeed, the sociopolitical context surrounding the COVID-19 pandemic in the U.S. was in many ways unique, and these factors are likely to have shaped some of our effects. In particular, as discussed above, the stark political polarization surrounding the pandemic in the U.S. is likely to have been at least partially responsible for the inflated ideological differences in self-reported (versus behavioral) responses to the pandemic. Future research will need to examine the degree to which these processes extend beyond the U.S. to other nations and cultural contexts.

## Concluding remarks

In this research, we provided a critical reexamination of the interrelations among disgust sensitivity, political ideology, and pandemic response. These results present an intriguing portrait, confirming some past findings, challenging others, and suggesting important caveats for yet others. Although many questions remain, we believe that this work sheds important light on several areas of research, including the relation between disgust sensitivity and ideology, and between disgust/BIS activation and disease avoidance. Taken together, this work also helps to shed light upon the conservatism-disgust paradox—the intriguing and seemingly contradictory pattern of relations that spurred our interest in these questions—by suggesting that the true pattern of interrelations is not as paradoxical as typically believed. We hope that these ideas will prove generative for future research, both that which seeks to understand responses to the current pandemic, as well as that which aims to deepen our understanding of the nature of, and relations among, these constructs.

## Supporting information

S1 File Key measures and additional analyses. (DOCX)
