# Supplementary material for: Investigating the conservatism-disgust paradox in reactions to the COVID-19 pandemic: A reexamination of the interrelations among political ideology, disgust sensitivity, and pandemic response
Source: PLoS One. 2022 Nov 4;17(11):e0275440. doi: 10.1371/journal.pone.0275440 (PMC9635700; doi:10.1371/journal.pone.0275440)
Supplement: S1 File — (DOCX) [file pone.0275440.s001.docx]

**Supporting Information**

**Key Measures and Additional Analyses**

Below are the key measures and additional analysis details for our studies. Variable names are included in brackets and correspond to those used in the data and syntax files. The full materials and measures for all studies—including the image stimuli not contained in this document—are available on the OSF page at https://osf.io/3ypx2/?view_only=dec5abe4eed6465bae473b3a96992b6d.

**STUDY 1**

**Disgust Sensitivity**

Disgust Scale-Revised (Haidt et al., 1994; Olatunji et al., 2007)

Please indicate how much you agree with each of the following statements, or how true it is about you.

0 = Strongly disagree (very untrue about me)

1 = Mildly disagree (somewhat untrue about me)

2 = Neither agree nor disagree

3 = Mildly agree (somewhat true about me)

4 = Strongly agree (very true about me)

[DSR1] I might be willing to try eating monkey meat, under some circumstances.

[DSR2] It would bother me to be in a science class, and to see a human hand preserved in a jar.

[DSR3] It bothers me to hear someone clear a throat full of mucus.

[DSR4] I never let any part of my body touch the toilet seat in public restrooms.

[DSR5] I would go out of my way to avoid walking through a graveyard.

[DSR6] Seeing a cockroach in someone else's house doesn't bother me.

[DSR7] It would bother me tremendously to touch a dead body.

[DSR8] If I see someone vomit, it makes me sick to my stomach.

[DSR9] I probably would not go to my favorite restaurant if I found out that the cook had a cold.

[DSR10] It would not upset me at all to watch a person with a glass eye take the eye

out of the socket.

[DSR11] It would bother me to see a rat run across my path in a park.

[DSR12] Even if I was hungry, I would not drink a bowl of my favorite soup if it had been stirred by a used but thoroughly washed flyswatter.

[DSR13] It would bother me to sleep in a nice hotel room if I knew that a man had died of a heart attack in that room the night before.

How disgusting would you find each of the following experiences?

0 = Not disgusting at all

1 = Slightly disgusting

2 = Moderately disgusting

3 = Very disgusting

4 = Extremely disgusting

[DSR14] You see maggots on a piece of meat in an outdoor garbage pail.

[DSR15] While you are walking through a tunnel under a railroad track, you smell urine.

[DSR16] You take a sip of soda, and then realize that you drank from the glass that an acquaintance of yours had been drinking from.

[DSR17] Your friend's pet cat dies, and you have to pick up the dead body with your bare hands.

[DSR18] You see someone put ketchup on vanilla ice cream, and eat it.

[DSR19] You see a man with his intestines exposed after an accident.

[DSR20] You discover that a friend of yours changes underwear only once a week.

[DSR21] A friend offers you a piece of chocolate shaped like dog‑doo.

[DSR22] You accidentally touch the ashes of a person who has been cremated.

[DSR23] You are about to drink a glass of milk when you smell that it is spoiled.

[DSR24] As part of a sex education class, you are required to inflate a new unlubricated condom, using your mouth.

[DSR25] You are walking barefoot on concrete, and you step on an earthworm.

**Pandemic Response**

[Worried] “Generally speaking, how worried are you that you personally will contract COVID-19 / the coronavirus?” (1 Not worried at all – 7 Extremely worried)

[SupportOpposeRule] “Do you support or oppose the guideline to engage in social distancing?”(-3 Strongly Oppose to +3 Strongly Support)

[SelfDistancing] “Generally speaking, how strictly have you personally been following the "social distancing" recommendations of the government and CDC to maintain a distance of six feet or more from others?”(7-point scale from “1 I’m not following these recommendations at all” to “7 I’m following these recommendations very strictly”

[HowOftenWashBefore] “How often are you now washing your hands or using hand sanitizer during a typical day compared to before the pandemic?”(7-point scale from “1 The same amount as before the pandemic” to “7 Much more than before the pandemic”)

**Political Orientation**

[PolOrient] Political orientation was assessed by the item: “Please select the scale point that best reflects your political orientation,” measured on a 7-point scale from “Extremely liberal” to “Extremely conservative.”

**Attention Check**

[attncheck] To ensure that participants were attending to the study, we included an attention check question: “Bob had a great day. He saw a beautiful butterfly. What did Bob see?” Four response options were provided: “a girl”, “a day”, “a fruit”, “an insect.” One participant chose an answer other than “an insect” and was therefore excluded from analyses.

**STUDY 2**

**Disgust Sensitivity**

Disgust Scale-Revised (Haidt et al., 1994; Olatunji et al., 2007)

Please indicate how much you agree with each of the following statements, or how true it is about you. Please write a number (0-4) to indicate your answer:

0 = Strongly disagree (very untrue about me)

1 = Mildly disagree (somewhat untrue about me)

2 = Neither agree nor disagree

3 = Mildly agree (somewhat true about me)

4 = Strongly agree (very true about me)

1. I might be willing to try eating monkey meat, under some circumstances.
2. It would bother me to be in a science class, and to see a human hand preserved in a jar.
3. It bothers me to hear someone clear a throat full of mucus.
4. I never let any part of my body touch the toilet seat in public restrooms.
5. I would go out of my way to avoid walking through a graveyard.
6. Seeing a cockroach in someone else's house doesn't bother me.
7. It would bother me tremendously to touch a dead body.
8. If I see someone vomit, it makes me sick to my stomach.
9. I probably would not go to my favorite restaurant if I found out that the cook had a cold.
10. It would not upset me at all to watch a person with a glass eye take the eye
11. out of the socket.
12. It would bother me to see a rat run across my path in a park.
13. Even if I was hungry, I would not drink a bowl of my favorite soup if it had been
14. stirred by a used but thoroughly washed flyswatter.
15. It would bother me to sleep in a nice hotel room if I knew that a man had died of a
16. heart attack in that room the night before.

How disgusting would you find each of the following experiences?

0 = Not disgusting at all

1 = Slightly disgusting

2 = Moderately disgusting

3 = Very disgusting

4 = Extremely disgusting

1. You see maggots on a piece of meat in an outdoor garbage pail.
2. While you are walking through a tunnel under a railroad track, you smell urine.
3. You take a sip of soda, and then realize that you drank from the glass that an acquaintance of yours had been drinking from.
4. Your friend's pet cat dies, and you have to pick up the dead body with your bare hands.
5. You see someone put ketchup on vanilla ice cream, and eat it.
6. You see a man with his intestines exposed after an accident.
7. You discover that a friend of yours changes underwear only once a week.
8. A friend offers you a piece of chocolate shaped like dog‑doo.
9. You accidentally touch the ashes of a person who has been cremated.
10. You are about to drink a glass of milk when you smell that it is spoiled.
11. As part of a sex education class, you are required to inflate a new unlubricated condom, using your mouth.
12. You are walking barefoot on concrete, and you step on an earthworm.

**Pandemic Response Measures**

To account for the changing context as the pandemic continued, we made some small changes to our dependent measures across the two study waves. For the second wave of Study 2, one attitudes item was dropped (COVIDConcern). To calculate the overall reliability of the attitudes measures, we calculated the reliability of the six measures included in Wave 1 (α = .73) and the reliability of the five items included in Wave 2 (α = .71), and then computed the mean of these two alphas: .72.

For the self-reported behavior items, ContactYesterday was included only in the first wave of Study 1, while SelfDistancing was included only in the second wave. Thus, participants always completed four items assessing self-reported behavior, but one item differed as a function of wave. To calculate the overall reliability of the self-reported behavior measure, we simply calculated the reliability of the four measures in Wave 2 (α = .63) and the reliability of the four measures included in Wave 4 (α = .79), and then computed the mean of these two alphas: .70.

**Attitude Measures**

The attitudes component of our composite pandemic response measure was comprised of the items listed below.

*Both Waves:*

[Worried] “Generally speaking, how worried are you that you personally will contract COVID-19 / the coronavirus?” (1 Not worried at all – 7 Extremely worried)

[Likely] “Generally speaking, how likely do you think it is that you personally will contract COVID-19 / the coronavirus?” (1 Very unlikely – 7 Extremely likely)

[COVIDExaggerated] “Do you believe the threat of COVID-19 / the coronavirus has been exaggerated?” (4-point scale from “Yes, I believe the threat has been greatly exaggerated” to “No, I believe the threat has not been conveyed strongly enough”)

[EconVsCOVID] “Authorities have closed businesses and recommended strict "social-distancing" to keep people safe from COVID-19. However, many experts predict that the economy will suffer because of these regulations. In your personal opinion, how should authorities weigh these two concerns?” (6-point scale from “Authorities should ONLY focus on protecting people from COVID-19 / the coronavirus, regardless of how much the economy will suffer.” to “Authorities should ONLY focus on protecting the economy, regardless of how many people will suffer from COVID-19 / the coronavirus.”)

[SupportOpposeRule] “Do you support or oppose the guideline to engage in social distancing?”(-3 Strongly Oppose to +3 Strongly Support)

*Wave 1 only*:

[COVIDConcern] “How concerned are you about the spread of COVID-19 / the coronavirus?” (“1 Not concerned at all” to “5 Very concerned”)

**Self-Reported Behavior Measures**

The self-reported behavior component of our composite pandemic response measure was comprised of the items listed below.

*Both Studies*:

[HowOftenWashBefore] “How often are you now washing your hands or using hand sanitizer during a typical day compared to before the pandemic?”(7-point scale from “1 The same amount as before the pandemic” to “7 Much more than before the pandemic”)

[UseFaceMask] “How regularly do you wear a face mask or face covering any time you leave your home?”(6-point scale from “Never/Do not have one” to “All of the time”)

[SelfQuarantine] In Studies 1-3, participants were asked “Generally speaking, how strictly have you personally been following the "social distancing" recommendations of the government and CDC (for example, staying home except for absolute necessities, having no contact with other people outside your household, etc.)” In Study 4, to reflect recent changes to the social-distancing guidelines in the U.S., participants were asked “Up to a few weeks ago, most people were under “shelter-in-place” orders (for example, staying home except for absolute necessities, having no contact with other people outside your household, etc.). During that time, how strictly did you follow these recommendations?” Both questions were assessed on a 7-point scale from “1 I did not follow these recommendations at all” to “7 I followed these recommendations very strictly.”

*Wave 1 only*:

[ContactYesterday] “Not counting the members of your household, approximately how many people did you have "close contact" with yesterday? ("close contact" means being closer than 6 feet away, even if it was only briefly)”(7-options: “zero”, “one”, “two”, “3-5”, “5-10”, “10-20”, “More than 20”)

*Wave 2 only*:

[SelfDistancing] “Generally speaking, how strictly have you personally been following the "social distancing" recommendations of the government and CDC to maintain a distance of six feet or more from others?”(7-point scale from “1 I’m not following these recommendations at all” to “7 I’m following these recommendations very strictly”

**Virtual Social Distancing Measures**

The virtual social distancing component of our composite pandemic response measure was comprised of the ten items described below. Question text and screenshots of each item are provided. The interactive version of each measure can be found at <http://psychvault.org/social-distancing-measures/>


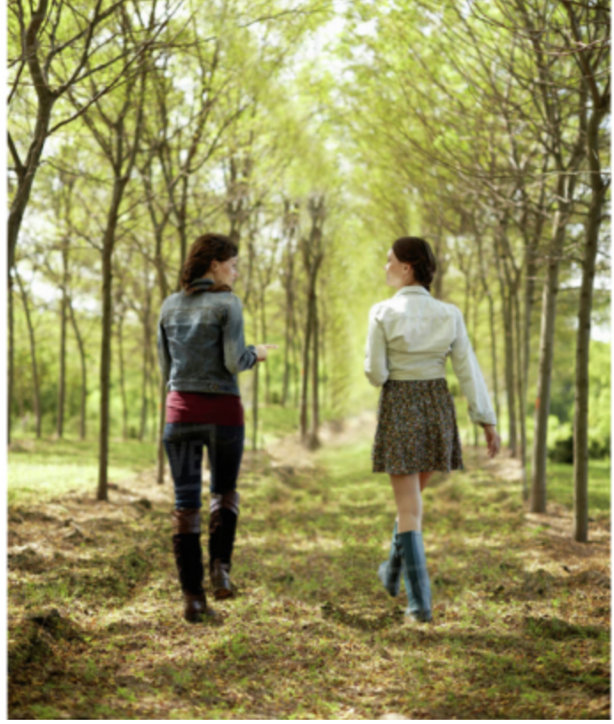


[TwoFriendsWoods] “Imagine you are out for a walk with a friend. What is the minimum distance you would feel comfortable having between the two of you?” This item was measured on an 11-point slider scale, corresponding to images depicting different distances between the two women.


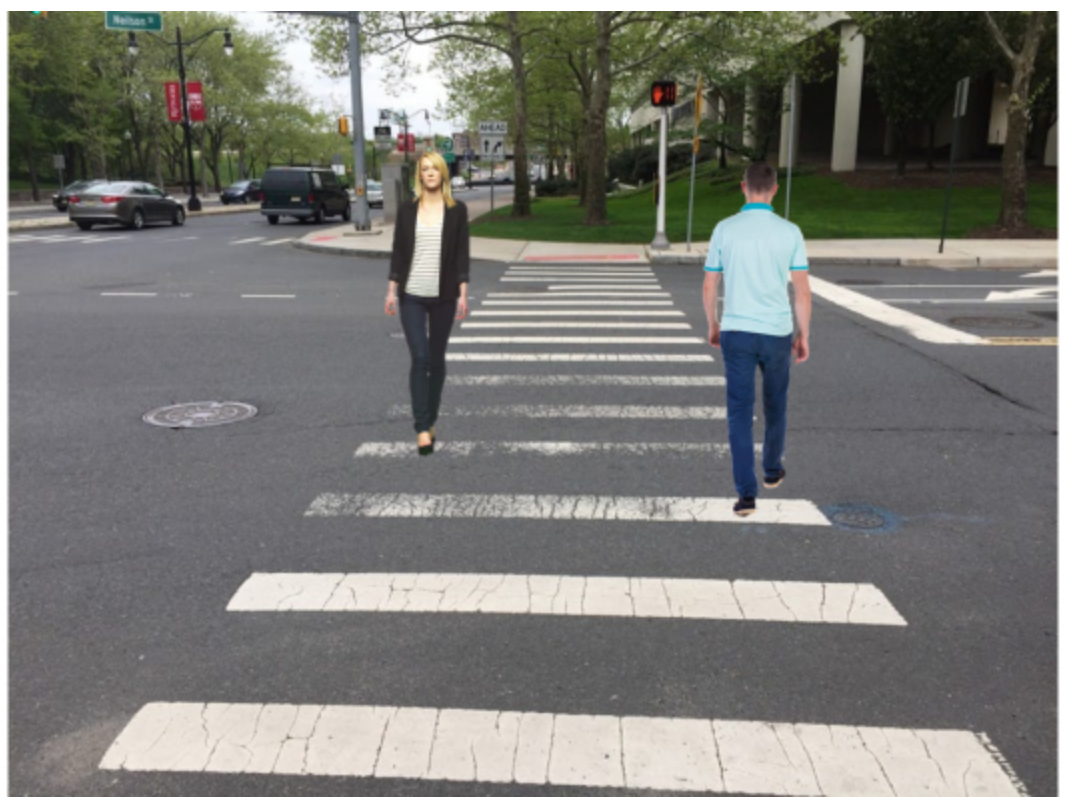


[CrosswalkDistance] “Imagine you are walking across the street and you pass someone walking the other way. How much distance would you leave between you?” This item was measured on an 11-point slider scale, corresponding to images depicting different distances between the two people.


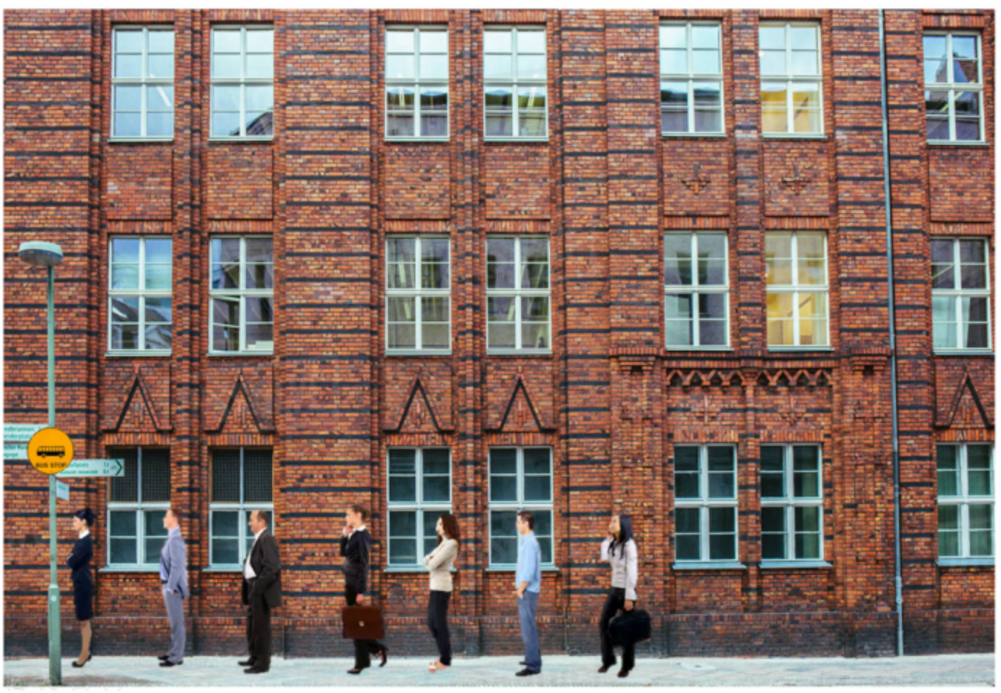


[StandInLineDistance] “Imagine you are standing in line waiting for the bus. How much distance would you want between you and the other people in line?” This item was measured on an 11-point slider scale, corresponding to images depicting different distances between the people.


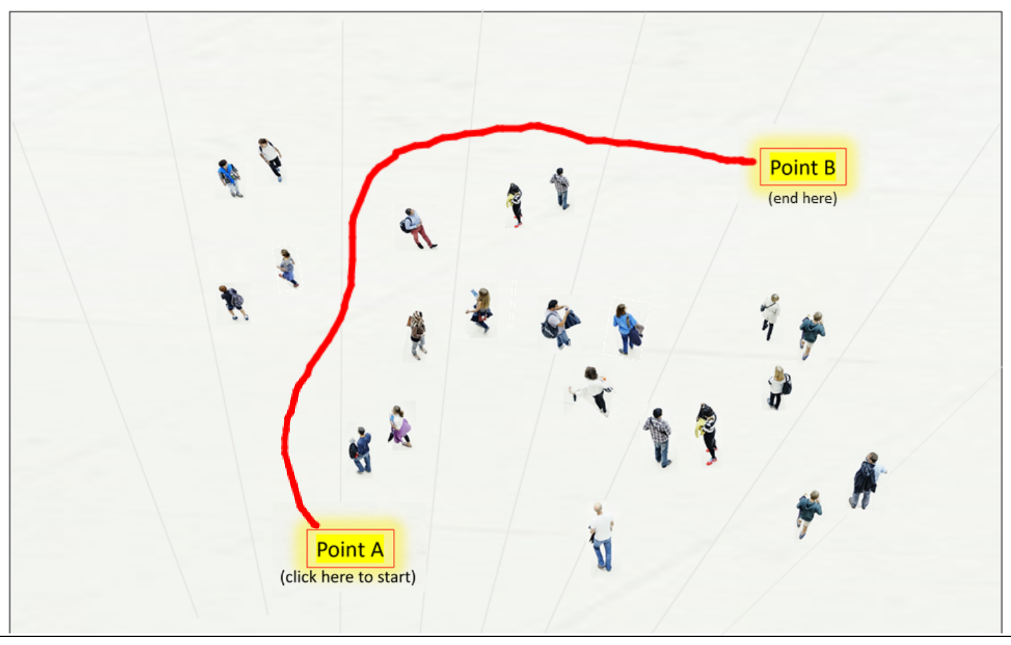


[crowd_path_length] “Imagine that you need to get to the other side of a crowded plaza. What path would you take?” Participants were free to draw any path through the crowd. We then calculated the total distance traveled, in pixels, to determine whether people took a more direct-but-crowded, versus indirect-but-solitary, path. Because of a technical error, the data from this measure were not recorded in Study 3. The red line in the image below illustrates an example response.


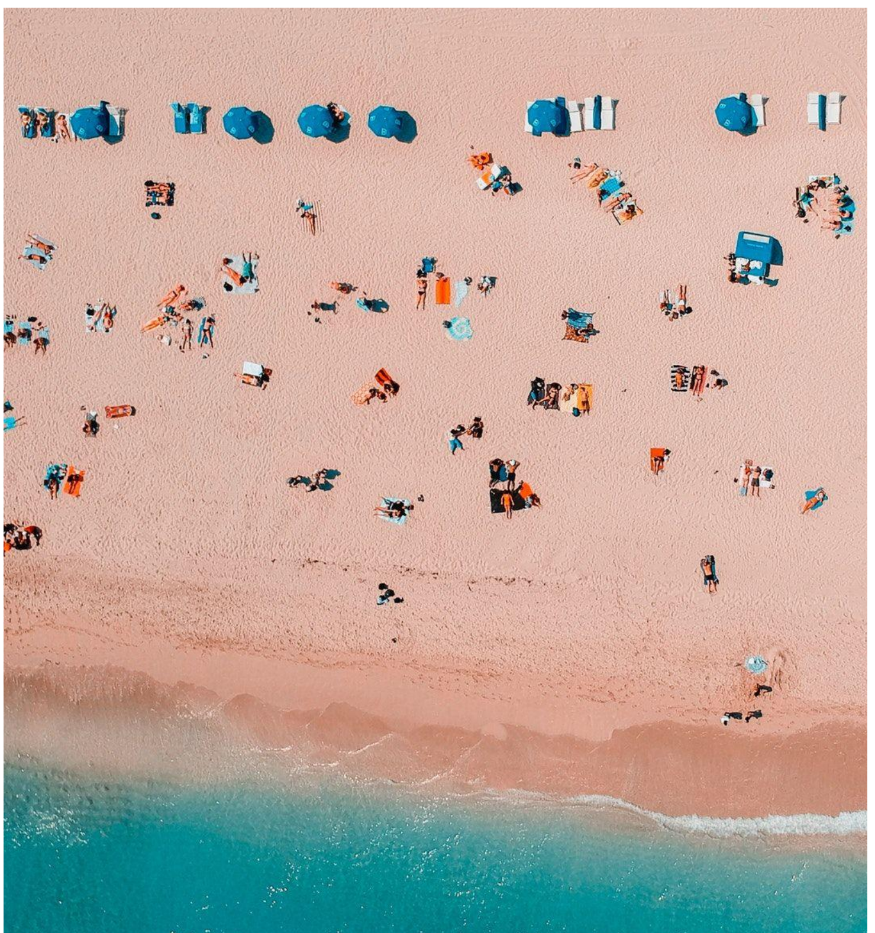


[beach_shortest_distance] “Click the point on the beach where you’d be most likely to lay down your towel.” Participants were free to choose any point on the beach. We then calculated the shortest distance, in pixels, between the point that the participant clicked and the nearest person on the beach.


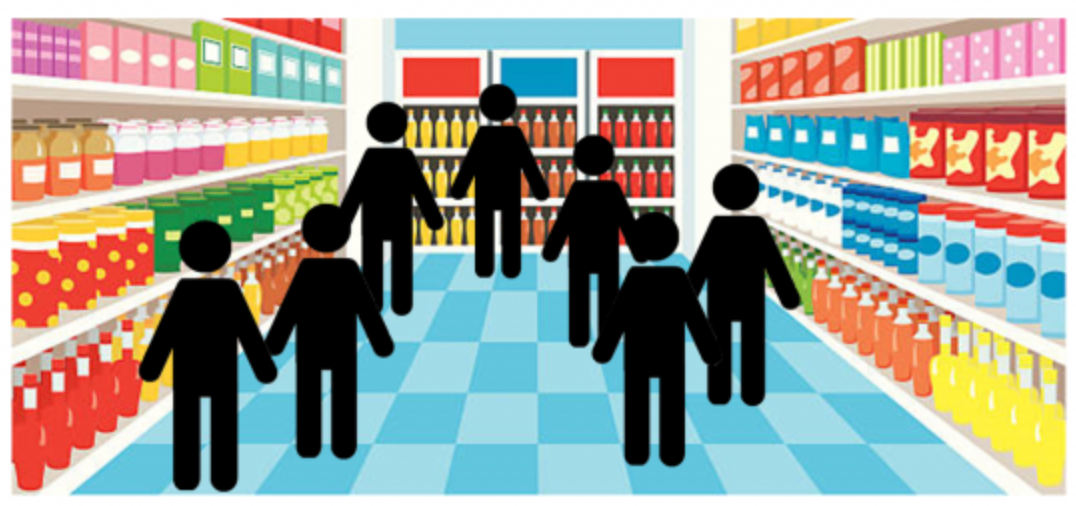
[GroceryStorePeople] “Here is a scene of a very crowded grocery store. Move the slider to “remove” people until you have reached the maximum number of people you would be comfortable having in the grocery store with you.” This item was measured on an 11-point slider scale, corresponding to images depicting different numbers of people.


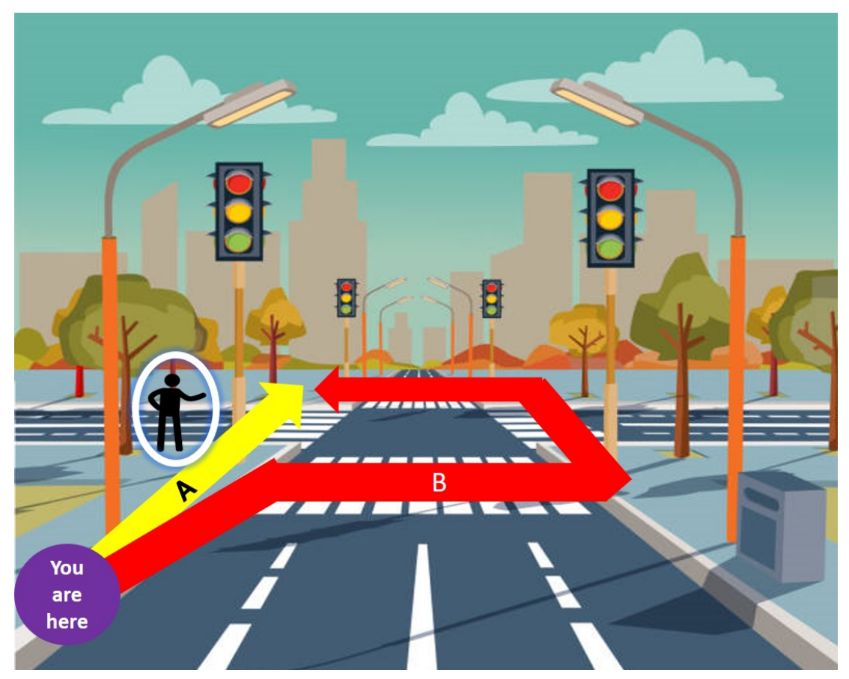


[TodayStreetPaths] “Imagine you are walking down the street when you see a person standing ahead of you at the corner. Do you continue on your path and walk directly by them (A), or do you go out of your way to avoid them (B)?” Responses were made on a 4-point scale: “I would definitely pick A”, “I would probably pick A”, “I would probably pick B”, “I would definitely pick B.”


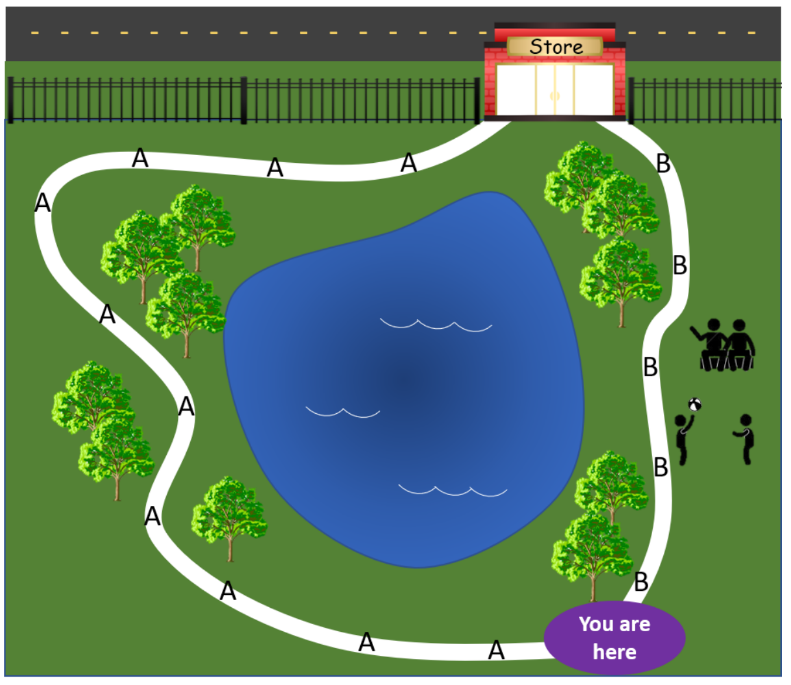
[TodayParkPaths] “Imagine you have to walk through a park to get to a store on the other side, and you have to choose one of two paths. One path through the park is typically quiet and secluded, but it is long (A). The other path typically has more people, but it is much shorter (B). Which path would you most likely choose?” Responses were made on a 4-point scale: “I would definitely pick A”, “I would probably pick A”, “I would probably pick B”, “I would definitely pick B.”


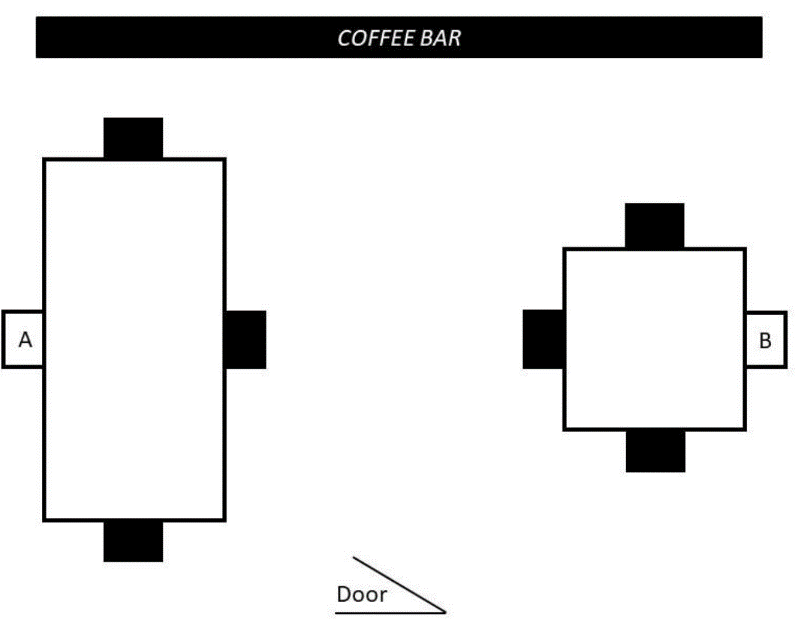


[FutureCoffee] “Imagine you walk into a coffee shop **that is once again open for business**, and you see there are only two open seats (A and B). Which seat would you most likely choose?” Responses were made on a 4-point scale: “I would definitely pick A”, “I would probably pick A”, “I would probably pick B”, “I would definitely pick B.”


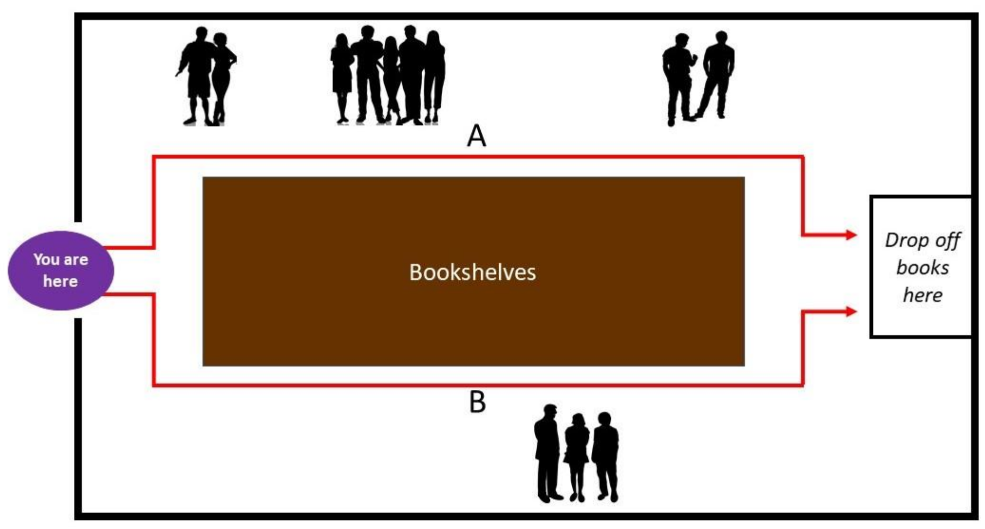


[FutureLibrary] “Imagine you need to return a book at the library **when it has reopened for business**. When you walk in, you see there are two equally direct paths to get to the drop-off site (A and B). Which path would you most likely choose?” Responses were made on a 4-point scale: “I would definitely pick A”, “I would probably pick A”, “I would probably pick B”, “I would definitely pick B.”

**Scoring of Dependent Measures**

To create our composite pandemic response measure, we first z-scored all items within study wave. To give equal weight to each of the three dimensions of pandemic response—attitudes, self-reported behavior, and virtual distancing—we first created separate composite scores for each of these three dimensions by calculating the mean of the standardized items in each category [composite variables are named MeanAttitudes, MeanSelfReportBehavior, and MeanBehavioralDistancing in the dataset]. Finally, we calculated the mean of these three composite scores to create our composite pandemic response measure [MeanOverallCOVIDResponse].

**Political Orientation**

[PolOrient] Political orientation was assessed by the item: “Please select the scale point that best reflects your political orientation,” measured on a 7-point scale from “Extremely liberal” to “Extremely conservative.”

**Attention Check**

[attncheck] As in Study 1, we included an attention check question: “Bob had a great day. He saw a beautiful butterfly. What did Bob see?” Four response options were provided: “a girl”, “a day”, “a fruit”, “an insect.” Participants who chose an answer other than “an insect” were excluded from analyses.

**STUDY 3**

**Experiential Disgust Sensitivity**

Participants rated four images that corresponded to stimuli described in the DS-R vignettes. They responded to the question “How disgusting do you find this image?” and rated each image on an 11-point scale from 0 “Not disgusting at all” to 10 “Extremely disgusting.”

**Item 1**:

DSR Vignette: “You see maggots on a piece of meat in an outdoor garbage pail.”

Image: Maggots on meat (see materials on OSF)

**Item 2**:

DSR vignette: “It would not upset me at all to watch a person with a glass eye take the eye out of the socket” (reverse-coded)

Image: A person taking a glass eye out of the socket (see materials on OSF)

**Item 3:**

DSR vignette: “It would bother me to be in a science class, and to see a human hand preserved in a jar.”

Image: A human hand preserved in a jar (see materials on OSF)

**Item 4**:

DSR vignette: “You see someone put ketchup on vanilla ice cream, and eat it.”

Image: ketchup on ice cream (see materials on OSF)

**Ambiguously/Mildly Disgusting Image**

Image of a cake shaped like a cockroach (see materials on OSF)

**Pandemic Response**

To determine which attitudes item had the highest item-total correlation, we analyzed the data from the second wave of Study 2, in which all six attitudes items were included. Based on this analysis, we selected this item:

[Worried] “Generally speaking, how worried are you that you personally will contract COVID-19 / the coronavirus?” (1 Not worried at all – 7 Extremely worried)

**STUDY 4**

In Study 4, we included a wider range of positive control images, as well as some additional—more mild—disgusting images (e.g., a cake shaped like raw chicken) to ensure that the lack of a relation between ideology and experiential disgust sensitivity could not explained by a ceiling effect for our experiential disgust measure (it was not; ratings of these mildly disgusting images was also not associated with ideology: β = .001, *t*(150) = 0.01, *p* = .99). Because of the range of image content we included, we modified the scale anchors to be “-5 Extremely Unappealing” and “+5 Extremely Appealing.”

**Self-Reported Disgust Sensitivity**

Disgust Scale-Revised (Haidt et al., 1994; Olatunji et al., 2007)

Please indicate how much you agree with each of the following statements, or how true it is about you.

0 = Strongly disagree (very untrue about me)

1 = Mildly disagree (somewhat untrue about me)

2 = Neither agree nor disagree

3 = Mildly agree (somewhat true about me)

4 = Strongly agree (very true about me)

[DSR1] I might be willing to try eating monkey meat, under some circumstances.

[DSR2] It would bother me to be in a science class, and to see a human hand preserved in a jar.

[DSR3] It bothers me to hear someone clear a throat full of mucus.

[DSR4] I never let any part of my body touch the toilet seat in public restrooms.

[DSR5] I would go out of my way to avoid walking through a graveyard.

[DSR6] Seeing a cockroach in someone else's house doesn't bother me.

[DSR7] It would bother me tremendously to touch a dead body.

[DSR8] If I see someone vomit, it makes me sick to my stomach.

[DSR9] I probably would not go to my favorite restaurant if I found out that the cook had a cold.

[DSR10] It would not upset me at all to watch a person with a glass eye take the eye

out of the socket.

[DSR11] It would bother me to see a rat run across my path in a park.

[DSR12] Even if I was hungry, I would not drink a bowl of my favorite soup if it had been stirred by a used but thoroughly washed flyswatter.

[DSR13] It would bother me to sleep in a nice hotel room if I knew that a man had died of a heart attack in that room the night before.

How disgusting would you find each of the following experiences?

0 = Not disgusting at all

1 = Slightly disgusting

2 = Moderately disgusting

3 = Very disgusting

4 = Extremely disgusting

[DSR14] You see maggots on a piece of meat in an outdoor garbage pail.

[DSR15] While you are walking through a tunnel under a railroad track, you smell urine.

[DSR16] You take a sip of soda, and then realize that you drank from the glass that an acquaintance of yours had been drinking from.

[DSR17] Your friend's pet cat dies, and you have to pick up the dead body with your bare hands.

[DSR18] You see someone put ketchup on vanilla ice cream, and eat it.

[DSR19] You see a man with his intestines exposed after an accident.

[DSR20] You discover that a friend of yours changes underwear only once a week.

[DSR21] A friend offers you a piece of chocolate shaped like dog‑doo.

[DSR22] You accidentally touch the ashes of a person who has been cremated.

[DSR23] You are about to drink a glass of milk when you smell that it is spoiled.

[DSR24] As part of a sex education class, you are required to inflate a new unlubricated condom, using your mouth.

[DSR25] You are walking barefoot on concrete, and you step on an earthworm.

**Experiential Disgust Sensitivity**

Participants rated four images that corresponded to stimuli described in the DS-R vignettes. They responded to the question “How appealing do you find this image?” and responded on an 11-point scale from -5 “Extremely unappealing” to +5 “Extremely appealing.”

**Item 1**:

DSR Vignette: “You see maggots on a piece of meat in an outdoor garbage pail.”

Image: Maggots on meat (see materials on OSF)

**Item 2**:

DSR vignette: “It would not upset me at all to watch a person with a glass eye take the eye out of the socket” (reverse-coded)

Image: A person taking a glass eye out of the socket (see materials on OSF)

**Item 3:**

DSR vignette: “It would bother me to be in a science class, and to see a human hand preserved in a jar.”

Image: A human hand preserved in a jar (see materials on OSF)

**Item 4**:

DSR vignette: “You see someone put ketchup on vanilla ice cream, and eat it.”

Image: ketchup on ice cream (see materials on OSF)

**Ambiguously/Mildly Disgusting Image**

Images of cut/sliced cakes shaped like (1) a cockroach, (2) a bulldog, (3) a raw turkey, (4) a human face (see materials on OSF)

**Pandemic Response**

In addition to the items used in our previous studies, this study also included some additional pilot questions relating to the COVID-19 pandemic (see OSF page). However, for consistency with our previous studies, and because several of these items showed little variance, for our analyses of Study 4 we include only the items that were included in our previous studies. However, the results do not meaningfully change if these items are included in our composite measures.

[Worried] “Generally speaking, how worried are you that you personally will contract COVID-19 / the coronavirus?” (1 Not worried at all – 7 Extremely worried)

[Likely] “Generally speaking, how likely do you think it is that you personally will contract COVID-19 / the coronavirus?” (1 Very unlikely – 7 Extremely likely)

[EconVsCOVID] “Authorities have closed businesses and recommended strict "social-distancing" to keep people safe from COVID-19. However, many experts predict that the economy will suffer because of these regulations. In your personal opinion, how should authorities weigh these two concerns?” (6-point scale from “Authorities should ONLY focus on protecting people from COVID-19 / the coronavirus, regardless of how much the economy will suffer.” to “Authorities should ONLY focus on protecting the economy, regardless of how many people will suffer from COVID-19 / the coronavirus.”)

[SelfQuarantine] In Studies 1-3, participants were asked “Generally speaking, how strictly have you personally been following the "social distancing" recommendations of the government and CDC (for example, staying home except for absolute necessities, having no contact with other people outside your household, etc.)” In Study 4, to reflect recent changes to the social-distancing guidelines in the U.S., participants were asked “Up to a few weeks ago, most people were under “shelter-in-place” orders (for example, staying home except for absolute necessities, having no contact with other people outside your household, etc.). During that time, how strictly did you follow these recommendations?” Both questions were assessed on a 7-point scale from “1 I did not follow these recommendations at all” to “7 I followed these recommendations very strictly.”

[ContactYesterday] “Not counting the members of your household, approximately how many people did you have "close contact" with yesterday? ("close contact" means being closer than 6 feet away, even if it was only briefly)”(7-options: “zero”, “one”, “two”, “3-5”, “5-10”, “10-20”, “More than 20”)

**Political Orientation**

[PolOrient] Political orientation was assessed by the item: “Please select the scale point that best reflects your political orientation,” measured on a 7-point scale from “Extremely liberal” to “Extremely conservative.”

**Appendix: Regression Results for All Studies**

| IV | DV | | β | df | *t* | *p* | CI LB | CI UB |
| --- | --- | --- | --- | --- | --- | --- | --- | --- |
|  | **STUDY 1** |  | |  |  |  |  |  |
| Self-Report Disgust | Conservatism | 0.17 | | 297 | 2.99 | 0.003 | 0.06 | 0.28 |
| Self-Report Disgust | Overall Pandemic Response | 0.20 | | 297 | 3.59 | <.001 | 0.09 | 0.32 |
| Conservatism | Overall Pandemic Response | -0.32 | | 297 | -5.81 | <.001 | -0.43 | -0.21 |
|  |  |  | |  |  |  |  |  |
|  | **STUDY 2** |  | |  |  |  |  |  |
| Self-Report Disgust | Conservatism | 0.18 | | 896 | 5.38 | <0.01 | 0.11 | 0.24 |
| Self-Report Disgust | Overall Pandemic Response | 0.24 | | 898 | 7.44 | <.001 | 0.18 | 0.30 |
| Conservatism | Overall Pandemic Response | -0.33 | | 897 | -10.43 | <.001 | -0.39 | -0.27 |
| Self-Report Disgust | Self-Report Pandemic Response | 0.26 | | 898 | 7.99 | <.001 | 0.19 | 0.32 |
| Self-Report Disgust | Behavioral Pandemic Response | 0.13 | | 898 | 3.91 | <.001 | 0.06 | 0.19 |
| Conservatism | Self-Report Pandemic Response | -0.33 | | 897 | -10.58 | <.001 | -0.40 | -0.27 |
| Conservatism | Behavioral Pandemic Response | -0.22 | | 897 | -6.69 | <.001 | -0.28 | -0.15 |
| Conservatism | Self-Report Pandemic Attitudes | -0.36 | | 897 | -11.57 | <.001 | -0.42 | -0.30 |
| Conservatism | Self-Report Pandemic Behavior | -0.25 | | 897 | -7.82 | <.001 | -0.32 | -0.19 |
|  |  |  | |  |  |  |  |  |
|  | **STUDY 3** |  | |  |  |  |  |  |
| Experiential Disgust | Self-Report Pandemic Response | 0.13 | | 295 | 2.22 | .03 | 0.02 | 0.24 |
| Ambiguous Image | Self-Report Pandemic Response | 0.19 | | 295 | 3.28 | .001 | 0.08 | 0.30 |
| Experiential Disgust w/ Positive Control | Self-Report Pandemic Response | 0.11 | | 294 | 2.05 | .04 | 0.004 | 0.22 |
|  |  |  | |  |  |  |  |  |
|  | **STUDY 4** |  | |  |  |  |  |  |
| Self-Report Disgust | Conservatism | 0.17 | | 150 | 2.14 | 0.03 | 0.01 | 0.33 |
| Self-Report Disgust | Overall Pandemic Response | 0.16 | | 150 | 1.95 | .053 | -0.002 | 0.32 |
| Conservatism | Overall Pandemic Response | -0.22 | | 150 | -2.75 | .007 | -0.38 | -0.06 |
| Experiential Disgust | Overall Pandemic Response | 0.26 | | 150 | 3.30 | .001 | 0.10 | 0.42 |
| Experiential Disgust w/ Self-Report Disgust Control | Overall Pandemic Response | 0.23 | | 149 | 2.76 | .007 | 0.06 | 0.40 |
| Self-Report Disgust w/ Experiential Disgust Control | Overall Pandemic Response | 0.07 | | 149 | 0.85 | .40 | -0.10 | 0.24 |
| Experiential Disgust w/ Self-Report Disgust Control (4 DS-R Vignettes Only) | Overall Pandemic Response | 0.24 | | 149 | 2.90 | .004 | 0.08 | 0.40 |
| Self-Report Disgust w/ Experiential Disgust Control (4 DS-R Vignettes Only) | Overall Pandemic Response | 0.08 | | 149 | 0.91 | .37 | -0.09 | 0.24 |
| Experiential Disgust w/ Self-Report Disgust Control | Behavioral Pandemic Response | 0.23 | | 149 | 2.74 | .007 | 0.07 | 0.40 |
| Self-Report Disgust w/ Experiential Disgust Control | Behavioral Pandemic Response | 0.03 | | 149 | 0.40 | .69 | -0.13 | 0.20 |
| Experiential Disgust w/ Self-Report Disgust Control | Self-Report Pandemic Response | 0.16 | | 149 | 1.81 | .07 | -0.01 | 0.33 |
| Self-Report Disgust w/ Experiential Disgust Control | Self-Report Pandemic Response | 0.08 | | 149 | 0.90 | .37 | -0.09 | 0.25 |
| Experiential Disgust | Conservatism | -0.10 | | 150 | -1.24 | 0.22 | -0.26 | 0.06 |
| Mild Disgusting Images | Conservatism | -0.02 | | 150 | -0.19 | 0.85 | -0.18 | 0.15 |
|  |  |  | |  |  |  |  |  |
